# Neuronal extracellular microRNAs miR‐124 and miR‐9 mediate cell–cell communication between neurons and microglia

**DOI:** 10.1002/jnr.24344

**Published:** 2018-10-27

**Authors:** Tatyana Veremeyko, Inna S. Kuznetsova, Marina Dukhinova, Amanda W. Y. Yung, Ekaterina Kopeikina, Natasha S. Barteneva, Eugene D. Ponomarev

**Affiliations:** ^1^ School of Biomedical Sciences The Chinese University of Hong Kong Shatin Hong Kong; ^2^ Program in Cellular and Molecular Medicine, Children’s Hospital Boston and Department of Pediatrics Harvard Medical School Boston Massachusetts; ^3^ School of Science and Technology Nazarbayev University Astana Kazakhstan; ^4^ Kunming Institute of Zoology Chinese University of Hong Kong Joint Laboratory of Bioresources and Molecular Research of Common Diseases Kunmin China

**Keywords:** extracellular miRNA, HDL, macrophages, microglia, miR‐124

## Abstract

In contrast to peripheral macrophages, microglia in the central nervous system (CNS) exhibit a specific deactivated phenotype; however, it is not clear how this phenotype is maintained. Two alternative hypotheses were postulated recently: (a) microglia differ from peripheral macrophages being derived from the yolk sac (YS), whereas peripheral macrophages originate from bone marrow (BM); (b) microglia acquire a specific phenotype under the influence of the CNS microenvironment. We have previously shown that microglia express miR‐124, which was also induced in BM‐derived macrophages co‐cultured with a neurons. We here investigated the possibility of horizontal transfer of the neuron‐specific microRNAs miR‐124 and miR‐9 from primary neurons to microglia/macrophages. We found that after incubation with neuronal conditioned media (NCM), macrophages downregulated activation markers MHC class II and CD45. Neither cultured adult microglia nor YS‐ and BM‐derived macrophages demonstrated intrinsic levels of miR‐124 expression. However, after incubation with NCM, miR‐124 was induced in both YS‐ and BM‐derived macrophages. Biochemical analysis demonstrated that the NCM contained miR‐124 and miR‐9 in complex with small proteins, large high‐density lipoproteins (HDLs), and exosomes. MiR‐124 and miR‐9 were promptly released from neurons, and this process was inhibited by tetrodotoxin, indicating an important role of neuronal electric activity in secretion of these microRNAs. Incubation of macrophages with exogenous miR‐124 resulted in efficient translocation of miR‐124 into the cytoplasm. This study demonstrates an important role of neuronal miRNAs in communication of neurons with microglia, which favors the hypothesis that microglia acquire a specific phenotype under the influence of the CNS microenvironment.

**Significance**

In our study, we demonstrated phenomenon of the secretion of extracellular microRNAs by electrically active neurons. Neuronal microRNAs were present in the extracellular space in various forms such as in complex with high‐density lipoproteins (HDLs). HDL:miRNA complexes were efficiently transferred from the extracellular space into the cytoplasm of macrophages. Horizontal transfer of miR‐124 from neurons to microglia was also detected in vivo. Our study demonstrates that microglia/macrophages acquire a specific phenotype only under the influence of the CNS microenvironment regardless of their origin from bone marrow or yolk sac, a finding that may have future clinical applications to downmodulate neuroinflammation.

## 1 INTRODUCTION

It is currently accepted that the central nervous system (CNS) possesses a specialized immunosuppressive microenvironment for immune cells such as macrophages, which often demonstrate a low level of activation in the normal CNS and even during various pathologies such as neuroinflammation. Within the CNS, the immune response is typically self‐limited when compared to other organs in the periphery (Fabry, Schreiber, Harris, & Sandor, 2008; Ponomarev, Maresz, Tan, & Dittel, 2007; Ponomarev, Veremeyko, & Weiner, 2013). Microglia are CNS‐resident immune cells that belong to the class of tissue‐resident macrophages and exhibit a deactivated phenotype in the normal CNS with low levels of activation markers MHC class II and CD45 (Ponomarev, Shriver, Maresz, & Dittel, 2005). The mechanism involved in such deactivation of CNS‐resident macrophages is still largely unknown. In our previous studies, we found that only microglia (but not other types of tissue‐resident or blood‐derived macrophages) expressed neuronal microRNA miR‐124, which mediated their deactivated MHC class II^low^CD45^low^ phenotype (Ponomarev, Veremeyko, Barteneva, Krichevsky, & Weiner, 2011). However, it remains unknown how the expression of miR‐124 is induced in microglia. We hypothesized that specific signals from neuronal cells induce miR‐124 in microglia, but it was not known how it was mediated at the molecular level (Ponomarev, Veremeyko, Barteneva et al., 2011; Ponomarev et al., 2013).

MicroRNAs are small (<25 nucleotides long) non‐coding RNAs that mediate many essential processes such as cell proliferation, differentiation, and modulation of activation of innate immune cells (Ponomarev et al., 2013). Because we found that neuronal microRNA‐124 is expressed in CNS‐resident microglia but not in peripheral macrophages (Ponomarev, Veremeyko, Barteneva et al., 2011), we hypothesize that there is a horizontal transfer of neuronal microRNAs from neurons to microglia. For transfer of miRNA‐124 from one cell to another within the CNS, these miRNAs should be relocated from the cytoplasm of a donor cell into the extracellular space, and then transported from the extracellular space into the cytoplasm of the recipient cell. Indeed, it was discovered that extracellular microRNAs (ECmiRNAs) are present in many biological liquids such as plasma, cerebrospinal fluid, saliva, and tears. There are three main known pathways of ECmiRNA secretion: (a) exosomes, (b) miRNA:Ago2 complexes, and (c) miRNA:HDL complexes (Sohel, 2016).

In addition to miR‐124, the neuronal microRNA miR‐9 is expressed at a very high level in neurons (Coolen, Katz, & Bally‐Cuif, 2013). Both miR‐124 and miR‐9 are predominantly expressed by neurons but not glial cells (Coolen et al., 2013; Ponomarev, Veremeyko, Barteneva et al., 2011; Rao et al., 2016). MiR‐9 regulates many processes of neurogenesis via targeting important transcription factors such as *Foxgp1*, *Foxp1*, *and Foxp2 *(Radhakrishnan & Alwin Prem Anand, 2016)*. *Along with miR‐124, miR‐9 is a key regulator of neuronal differentiation (Krichevsky, Sonntag, Isacson, & Kosik, 2006). Both miR‐124a and miR‐9 negatively regulate phosphorylation of STAT3, which inhibits neuronal terminal differentiation (Krichevsky et al., 2006). Outside of the CNS, miR‐9 is expressed in lipopolysaccharide‐activated macrophages (Liu & Abraham, 2013) and is involved in regulation of expression of interferon‐regulated genes such as MHC class I, IL‐1, and IL‐6 (Gao et al., 2013). Thus, miR‐124 and miR‐9 could play an important role in modulating the functions of innate immune cells in the CNS microenvironment.

The most studied pathway is the secretion of miRNA via exosomes and other types of cell‐derived microparticles. Many types of miRNA have been found in small cell vesicles of 30–90 nm (exosomes), as well as larger (100–1,000 nm) microparticles that are produced by many cell types, including neurons (Barteneva et al., 2017, 2013 ; Zhang et al., 2015). Neuronal cells secrete a large number of exosomes, which originate from endosomes via an internal budding process and formation of multivesicular bodies comprising endosomes containing multiple exosome vesicles (Chivet, Hemming, Pernet‐Gallay, Fraboulet, & Sadoul, 2012; Zhang et al., 2015). Fusion of endosomes with the plasma membrane results in the release of multiple exosomes into the extracellular space. In neurons, endosomes are present in neuronal pre‐ and postsynaptic terminals (Cooney, Hurlburt, Selig, Harris, & Fiala, 2002). Postsynaptic neuronal terminals are enriched with multivesicular bodies that are increased by the synaptic (electric) activity of neurons (Kraev et al., 2009; Stewart et al., 2005). Secreted exosomes are taken up by other cells via endocytosis, where exosomes could fuse with endosomal or outer plasma membranes, leading to translocation of miRNA into the cytoplasm of recipient cells (Zhang et al., 2015). Thus, exosomes could serve as a “parcel service” for miRNA transfer among the cells.

In addition to secretion of ECmiRNA via exosomes, miRNAs have been found in the extracellular space in complex with an Ago2 protein and also in complex with high‐density lipoprotein (HDL) particles (Sohel, 2016). Ago2 is important for the chief functions of miRNA, namely RNA interference and gene silencing by forming RNA‐induced silencing complexes. In the cytoplasm, a large proportion of miRNA is in complex with Ago2. The mechanism of release of miRNA:Ago2 complexes is not clear (Sohel, 2016). Most likely these complexes are released from the cytoplasm into the extracellular space from necrotic or late apoptotic cells when the integrity of the plasma membrane becomes compromised (Turchinovich, Weiz, Langheinz, & Burwinkel, 2011) In contrast to Ago2, miRNA:HDL complexes are secreted by live cells under physiological conditions via an unknown mechanism. HDLs are abundant in the blood circulation and are also present in the CNS, where they are mostly produced by astrocytes and microglia (Vitali, Wellington, & Calabresi, 2014). MiRNA:HDL complexes are taken up by other cells such as macrophages or hepatocytes via scavenger SR‐BI receptors (Vickers, Palmisano, Shoucri, Shamburek, & Remaley, 2011). The mechanism of release from miRNA:HDL complexes is not clear, but it is known that miRNA could bind to the internal part of dense detergent‐resistant membrane domains (also referred to as lipid rafts) during the process of exosome budding (Janas, Janas, Sapoń, & Janas, 2015; Michell & Vickers, 2016; Vickers et al., 2011). Inhibition of the enzyme sphingomyelinase‐2, which cleaves glycosphingolipids in the areas of lipid rafts, increased the number of HDL:miRNA complexes (Michell & Vickers, 2016) and exosomes containing miRNA (Kosaka et al., 2010). MiRNA has the ability to bind glycolipids in complex with HDL via divalent cation binding (Janas et al., 2015; Janas, Janas, & Yarus, 2006; Khvorova, Kwak, Tamkun, Majerfeld, & Yarus, 1999). Thus, miRNA has the ability to bind to HDL‐associated lipids, as well as internal and external parts of cell plasma membranes, especially areas of glycosphingolipid‐enriched lipid rafts. Postsynaptic terminals are significantly enriched with neuronal lipid rafts found in the area of post‐synaptic density (Ponomarev, 2018; Sotnikov et al., 2013) and may also serve as an additional external site for miRNA binding for subsequent release into the extracellular space via shedding and/or enzymatic cleavage of glycosphingolipids (e.g., sphingomyelinase, β‐galactosidase) (Janas et al., 2015, 2006; Khvorova et al., 1999).

In this study, we tested our hypothesis that there is a horizontal transfer of neuron‐specific microRNAs miR‐124 and miR‐9 from neurons to microglia and macrophages in normal CNS, which determined deactivated phenotype of monocytic cells in CNS microenvironment. We also investigated possible molecular mechanism of this phenomenon.

## 2 MATERIALS AND METHODS

### 2.1 Mice

C57BL/6 (B6; RRID: IMSR_JAX:000664) and B6.ACTB‐DsRed (B6.Cg‐Tg(CAG‐DsRed*MST)1Nagy/J; RRID: IMSR_CMMR:0071) transgenic (DsRed tg) mice were originally purchased from the Jackson Laboratory (Bar Harbor, ME) and bred locally at the Laboratory Animal Services Center (LASEC) at the Chinese University of Hong Kong. The study was performed in accordance with the recommendations of the ARRIVE guidelines (https://www.nc3rs.org.uk/arrive-guidelines). Animal protocols were approved by the Department of Health of the Government of Hong Kong and the Chinese University of Hong Kong Animal Experimentation Ethics Committee. Animals were housed under strictly controlled environment (temperature and humidity) with automatic 12‐hr light/dark cycle and were fed ad libitum. Animals were provided with housing cages (4–5 animals of the same sex per cage) and bedding, which was changed daily. All materials and personnel were passed through a decontamination and/or sterilization process before entering animal facility. For all experimental procedures, we used both male and female mice of different age for different procedures, which is specified below. For preparation of bone marrow or brain slices the animals were sacrificed by CO_2_ overdose followed by cervical dislocation. For preparation of microglia or sections for immunofluorescene, mice were anasthesized with lethal dose of isofluran for the subsequent intracardial whole‐body perfusion with PBS. Mouse embrions were analgised by placing on ice and euthnized by swift decapitation.

### 2.2 Cells

Bone marrow‐derived macrophages (BMDMs) were grown in the presence of macrophage colony‐stimulating factor (M‐CSF), as described earlier in our studies (Ponomarev, Veremeyko, Barteneva et al., 2011; Veremeyko, Siddiqui, Sotnikov, Yung, & Ponomarev, 2013). Bone marrow from 6‐ to 8‐week‐old B6 mice (combining equal number of males and females; *n* = 3–4 males and *n* = 3–4 females per experiment) was isolated by flushing, and a single‐cell suspension was obtained by passing the cell suspension through a 70‐μm cell strainer (Falcon). Yolk sac‐derived macrophages (YSDMs) were grown in M‐CSF, much as with BMDMs. Briefly, yolk sacs were isolated from day 10–11 mouse embryos of both sexes (approximately 4–6 male and 4–6 female embryos per experiment), homogenized by passing through a 26 g needle of a 1‐ml syringe. The cell suspension was passed through a 70‐μm cell strainer and re‐suspended in culture medium. BMDMs and YSDMs were grown in DMEM culture media (Gibco) supplemented with 10% FBS (Gibco) and 10 ng/ml M‐CSF (R&D) for 5 days. Media were replaced after every 2–3 days. After 5 days, the cells were used for experiments. FACS analysis indicated a homogeneous population of YSDMs with 90%–95% F4/80‐positive macrophages (data not shown). Adult microglia were isolated from the CNS of equal numbers of male and female 6‐ to 7‐week‐old B6 mice (combining *n* = 3 males and *n* = 3 females per experiment) using a 40%/70% Percoll gradient and grown in the presence of M‐CSF (10 ng/ml) as described in our earlier studies (Dukhinova, Kopeikina, & Ponomarev, 2018; Ponomarev, Novikova, Maresz, Shriver, & Dittel, 2005). Mouse cortical neurons were prepared from prenatal day 17–18 embryos of both sexes (combining *n* = 4–6 males and *n* = 4–6 females per experiment) as also described in our previous studies (Wong et al., 2013). Neuronal cultures contained 85%–90% neurons, 10%–15% astroglia, and an undetectable number of microglia, as determined by staining for β3‐tubulin, GFAP, and CD11b or Iba1, respectively (see information about antibodies below). Brain slice organotypic cultures (thickness: 500 μm) were obtained from equal numbers of male and female B6 mice aged 4–5 weeks (combining *n* = 3–4 males and *n* = 3–4 females per experiment) and were treated similarly as described (Kellner et al., 2014). Cortical neuronal cells or brain slices were cultured using a neurobasal medium with B27 supplement, 0.5% glutamate, and 1% penicillin/streptomycin (no AraC treatment), and used at 10–14 days in vitro. Co‐culture of primary cortical neurons with BMDMs was performed in neurobasal medium in the presence of M‐CSF (10 ng/ml). Co‐culture of neurons with BMDM did not markedly reduce neuronal and macrophage viability, as determined by staining for the neuronal and macrophage markers β3‐tubulin and CD11b before and after co‐culture. The neuronal cell line N1E115 (mouse neuroblastoma) was purchased from ATTC (cat# CRL‐2263; RRID: CVCL_0451) and cultured in DMEM with 10% FBS. Cell viability was measured by a bioluminescent kit from Abcam (cat#ab65314).

In our study, we used the cells from both sexes, but we did not specifically investigate gender difference. To exclude sex as a biological variable for macrophages in culture, we combined equal (bone marrow) or comparable (yolk sac) numbers of males and females as a source of isolated cells.

### 2.3 Reagents

Proteinase K, β‐galactosidase, monensin, ionomycin, and sodium azide (NaN_3_) were purchased from Sigma. RNase was purchased from Thermo Fischer Scientific (cat# EN0531). Tetrodotoxin (TTX) was purchased from Abcam.

For treatment of NCM or brain slice conditioned medium (BSCM), proteinase K, and RNase were used at final concentrations of 10 U/ml and 100 μg/ml, respectively, for 1 hr at 37°C. Exosomes were isolated using total exosomes isolation kit from Invitrogen (cat#4478359), according to the manufacturer's recommendations.

For treatment of cultured neurons, NaN_3_ and monensin were used at final concentrations of 10 nM and 2 μM, respectively. RNase was used at a final concentration of 100 μg/ml. TTX was used at a final concentration of 1 μM as described earlier (Takada, Yanagawa, & Komatsu, 2005). β‐Galactosidase was used at a concentration of 10 U/ml, similarly as described in our earlier studies (Sotnikov et al., 2013).

### 2.4 Antibody characterization

Rat anti‐mouse monoclonal anti‐CD11b‐AF488 antibodies (clone M1/70) that recognize macrophages marker CD11b (Veremeyko et al., 2018) were purchased from BD Biosciences (0.2 mg/ml, dilution 1:100; RRID: AB_396784). Goat polyclonal anti‐human/rat/mouse antibodies for Iba1 (NP_116573.1; NP_001614.3) were purchased from BioRad (cat#AHP2024; 0.5 mg/ml, dilution 1:200; RRID: AB_2224406) and were used with secondary donkey anti‐goat IgG antibodies conjugated with AF488 (Jackson ImmunoResearch, cat#705‐546‐147, RRID: AB_2340430). Mouse monoclonal AF488‐conjugated antibodies for human/mouse/rat GFAP (clone GA5) were purchased from EMD Millipore (cat#mab3402X; 1 mg/ml, dilution 1:1,000; RRID: AB_11210273). Rabbit polyclonal anti‐human/mouse/rat antibodies for β3‐tubulin (cat#ab15708A4; 1 mg/ml, dilution 1:1,000; RRID: AB_10916541) were purchased from EMD Millipore. For more technical information regarding antibodies please refer to the Table 1.

**Table 1:** Antibody characterization

The name of the antibody (host species, clonality, specificity)	Immunogen (species recognized)	Description (manufacturer, catalog number, RRID, control conditions)	The initial concentration and dilution
Rat anti‐mouse monoclonal anti‐CD11b‐AF488 antibodies (clone M1/70)	Macrophages marker CD11b (mouse, human)	BD Biosciences, cat# 557672, RRID: AB_396784, control: absence of binding to CD11b‐negative cell subsets (lymphocytes) as determined by FACS	0.2 mg/ml, dilution 1:100
Goat polyclonal anti‐human/rat/mouse antibodies for Iba1	Iba1/AIF1 (C‐TERMINAL) NP_116573.1; NP_001614.3 (mouse, human, rat, pig)	BioRad, cat#AHP2024, RRID: AB_2224406, control: absence of binding to Iba‐negative cell subsets (neurons, astrocytes) as determined by immunofluorescence	0.5 mg/ml, dilution 1:200
Donkey polyclonal anti‐goat IgG antibodies conjugated with AF488	Goat IgG (H + L) (goat)	Jackson ImmunoResearch, cat# 705‐546‐147, RRID: AB_2340430, control: antibodies have minimal cross reactivity to chicken, guinea pig, Syrian hamster, horse, human, mouse, rabbit, and rat serum proteins	1 mg/ml, dilution 1:500
Mouse monoclonal AF488‐conjugated antibodies for human/mouse/rat GFAP (clone GA5)	Glial Fibrillary Acidic Protein **(**human, mouse, rat)	EMD Millipore, cat#mab3402X, RRID: AB_11210273, control: absence of binding to GFAP‐negative cell subsets (neurons, microglia) as determined by immunofluorescence	1 mg/ml, dilution 1:1,000
Rabbit polyclonal anti‐human/mouse/rat antibodies for β3‐tubulin	Beta III Tubulin (human, mouse, rat)	EMD Millipore, cat#ab15708A4; RRID: AB_10916541, control: absence of binding to β3‐tubulin ‐negative cell subsets (astrocytes, microglia) as determined by immunofluorescence	1 mg/ml, dilution 1:1,000
Rat monoclonal anti‐mouse MHC class II‐PE‐Cy5 (clone MS/114.15.2)	MHC Class II (I‐A/I‐E) (mouse)	eBioscience, cat# 14‐5321‐81, RRID: AB_467560**, **control: absence of binding to MHC class II‐negative cell subsets (T lymphocytes) as determined by FACS	0.2 mg/ml, dilution 1:200
Rat monoclonal anti‐CD45‐APC‐Cy7 (clone 30‐F11)	CD45 (mouse)	BioLegend; cat# 103115, RRID: AB_312980**,** control: absence of binding to CD45‐negative cells (astroglial cell line) as determined by FACS	0.2 mg/ml, dilution 1:100
Rat monoclonal anti‐F4/80‐AF488 (clone BM8)	F4/80 (mouse)	BioLegend, cat# 12312, RRID: AB_893479, control: absence of binding to F4/80‐negative cell subsets (lymphocytes) as determined by FACS	0.5 mg/ml, dilution 1:200
Rat monoclonal antibodies specific for mouse Fc receptors CD16/CD32 (clone 2.4G2)	CD16/CD32 (mouse)	BD Biosciences, cat# 553141, RRID: AB_394656, control: these are blocking antibodies	0.5 mg/ml, dilution 1:50
Rabbit polyclonal antibodies for human/mouse/rat HDLs	KLH conjugated synthetic peptide from a 41–125 amino acid sequence of human HDL (human, mouse, rat)	Biorbyt, cat#orb1003, RRID: AB_2737031**, **control: absence of non‐specific bands on the western blot	0.5 mg/ml, dilution 1:1,000
Rabbit monoclonal anti‐human/mouse/rat TSG101 antibodies	Synthetic peptide from a 1–100 amino acid sequence of human TSG101 (human, mouse, rat)	Abcam, cat#ab125011, RRID: AB_10974262, control: absence of non‐specific bands on the western blot	1 mg/ml, dilution 1:1,000

### 2.5 Flow cytometry and cell sorting

For analysis of BMDMs, the cells were obtained from DsRed tg mice and stained with rat anti‐mouse monoclonal anti‐MHC class II‐PE‐Cy5 (eBioscience; clone MS/114.15.2; 0.2 mg/ml, dilution 1:200; RRID: AB_467560), anti‐CD45‐APC‐Cy7, (BioLegend; clone 30‐F11; 0.2 mg/ml, dilution 1:100; RRID: AB_312980), and anti‐F4/80‐AF488 (BioLegend; clone BM8; 0.5 mg/ml, dilution 1:200; RRID: AB_893479) as described earlier (Ponomarev, Tarasenko, & Sapozhnikov, 2000; Starossom, Veremeyko, Dukhinova, Yung, & Ponomarev, 2014; Veremeyko et al., 2018). F4/80^+^DsRed^+ ^gated macrophages were sorted from neuronal co‐cultures or analyzed for the expression of MHC class II and CD45. Fc receptors were blocked with a monoclonal antibodies specific for mouse Fc receptors (clone 2.4G2; BD Biosciences; 0.5 mg/ml, dilution 1:50; RRID: AB_394656). For more technical information regarding antibodies please refer to the Table 1.

For analysis of expression of activation markers, we used an LSRFortressa cytometer (BD Biosciences) (Dukhinova et al., 2018). For cell sorting, we used FACSAria 3 (BD Biosciences) (Kopeikina, Dukhinova, & Ponomarev, 2018). For data analysis FlowJo (RRID: SCR_008520) software was used.

### 2.6 Biochemical characterization of neuronal conditioned medium

Neuronal conditioned medium (NCM) was collected from mouse cortical neurons cultured for 10 days. On day 11, neurons were placed in fresh neurobasal medium and cultured for 24 hr when the NCM was harvested. Similarly, BSCM was collected from brain slice organotypic cultures after 24 hr of culture in fresh neurobasal medium. After harvesting, NCM or BSCM were centrifuged at 3,000 rpm and then passed through a 0.2‐μm filter (low protein binding membrane; Millipore) to eliminate cell debris. NCM or BSCM were further fractionated by using protein fractionation membranes for 3, 10, 30, 50, and 100 kDa (“Centurion”, Millipore). For further analysis, fractionated NCM or BSCM were treated with proteinase K (24 hr, 37°C), or RNase (24 hr, 37°C). NCM were also filtered using a stainless steel filter with a set of 100‐, 50‐, and 25‐nm nylon membranes (Millipore).

### 2.7 Quantitative real‐time RT PCR

For the analysis of miR‐124, miR‐9, miR‐155, and other miRNA expressions (miR‐124*, miR‐9*, miR‐134, miR‐138), we used primers from Applied Biosystems and analyzed either by showing 40‐C_T_ data (for conditioned media) or using snoRNA‐55 for normalization and the ΔΔC_T_ method (for the cells) as described previously (Ponomarev, Veremeyko, Barteneva et al., 2011; Veremeyko, Starossom, Weiner, & Ponomarev, 2012).

### 2.8 Western blotting

Western blot analysis was performed according to a standard protocol as previously reported (Ponomarev, Veremeyko, Barteneva et al., 2011; Veremeyko et al., 2018). Rabbit polyclonal antibodies for human/mouse/rat HDLs (immunogen: KLH conjugated synthetic peptide from a 41–125 amino acid sequence of human HDL) were purchased from Biorbyt (cat#orb1003; 0.5 mg/ml, dilution 1:1,000; RRID: AB_2737031). Rabbit monoclonal anti‐human/mouse/rat TSG101 antibodies (immunogen: synthetic peptide from a 1–100 amino acid sequence of human TSG101) were purchased from Abcam (cat#ab125011; dilution 1:1,000; RRID: AB_10974262). For more technical information regarding antibodies please refer to the Table 1.

### 2.9 Exosome preparation

Exosome preparation was performed using a total exosome isolation kit from Invitrogen (cat#4478359) according to the manufacturer’ recommendations. This kit is optimized to isolate vesicles of sizes 30–150 nm from culture supernatant by precipitating exosomes via centrifugation at 10,000 g.

### 2.10 Fluorescent in situ microRNA hybridization (FISH) and immunofluorescence histology

Visualization of miR‐124 in macrophages and glial cells was performed by in situ hybridization as described (Planell‐Saguer, Rodicio, & Mourelatos, 2010; Ponomarev, Veremeyko, & Barteneva, 2011). BMDMs were seeded on glass slides and fixed in freshly prepared 4% paraformaldehyde (PFA) in PBS for 30 min and then stored in PBS until the FISH procedure.

Brains were isolated from male (*n* = 3) and female (*n* = 3) of 8‐ to 12‐week‐old C57BL/6 mice following isoflurane overdose and whole‐body perfusion with PBS and then with 1% PFA. Tissue was fixed in 1% PFA (24 hr) and 30% sucrose (3 days), then transferred into TissueTek and sectioned on a cryotome to prepare 10‐µm‐thick histology sections.

Biotin labeling LNA probe (Exiqon) for detection of microRNA‐124 (bio‐3′‐ATCAAGGTCCGCTGGAACA‐5′‐bio) was mixed with the following: 50% formamide, 10% dextran sulfate, 100 ng of miRNA in 2× SSC and applied to glass slides with fixed cells. Probe hybridization was performed at 60°C (melting temperature (*T_m_*) is 77°C), 15 min for cultured macrophage culture and 30 min for brain histology sections. Post‐hybridization washes consisted of the wash with 4× SSC and then with 2× SSC (5 min each at 60°C). The slides were then rinsed three times in TNT solution (0.1 M Tris‐HCl, 0.15 M NaCl) at room temperature, and then used for visualization using Cy3‐labeled TSA fluorescence detection systems according to the company's recommendation (PerkinElmer).

Subsequently, samples were processed for immunostaining to detect microglia (Iba1‐positive cells) and astrocytes (GFAP‐positive cells) in brain histology sections, or for labeling cell membranes of cultured macrophages. FITC‐labeled Cholera Toxin B subunit (CTB‐FITC; 1:100; Sigma) was used to label ganglioside GM1 on the cell membrane of cultured macrophages. All slides were counterstained with DAPI and mounted in an anti‐fade solution (Vectashield; Vector Laboratories, Burlingame, CA, USA). Image acquisition was performed using an Olympus FV1000‐ZCD Laser Scanning Confocal System (RRID: SCR_012365), with 60× objective, 1× and 2.5× magnification. Final image adjustments were performed using Adobe Photoshop CS2 (RRID: SCR_014199) and Image J (RRID: SCR_003070) software.

### 2.11 Statistical data analysis

The results are presented as mean or median ± standard deviation (*SD*). Unpaired Student's *t* test was used to determine significance between two independent groups. Paired Student's *t* test was used for the same samples before and after treatment or for the same sample. One‐way ordinary or repeated measures ANOVA with or without Bonferroni post hoc test were used to determine statistical significance for experiments with three or more experimental groups. *P* values of less than 0.05 were considered significant. SigmaPlot (RRID: SCR_003210) and GraphPad Prism (RRID: SCR_002798) programs were used for the creation of the graphs and performing statistical analysis. Cumulative (integral) area under miRNA expression curves was calculated using the specific script for Excel software. All initial samples were coded with numbers to ensure randomization and minimize subjective bias when performing further sample processing and analysis using real‐time RT PCR or flow cytometry.

## 3 RESULTS

### 3.1 Neuronal soluble factors deactivate macrophages and upregulate miR‐124

**Figure 1:**
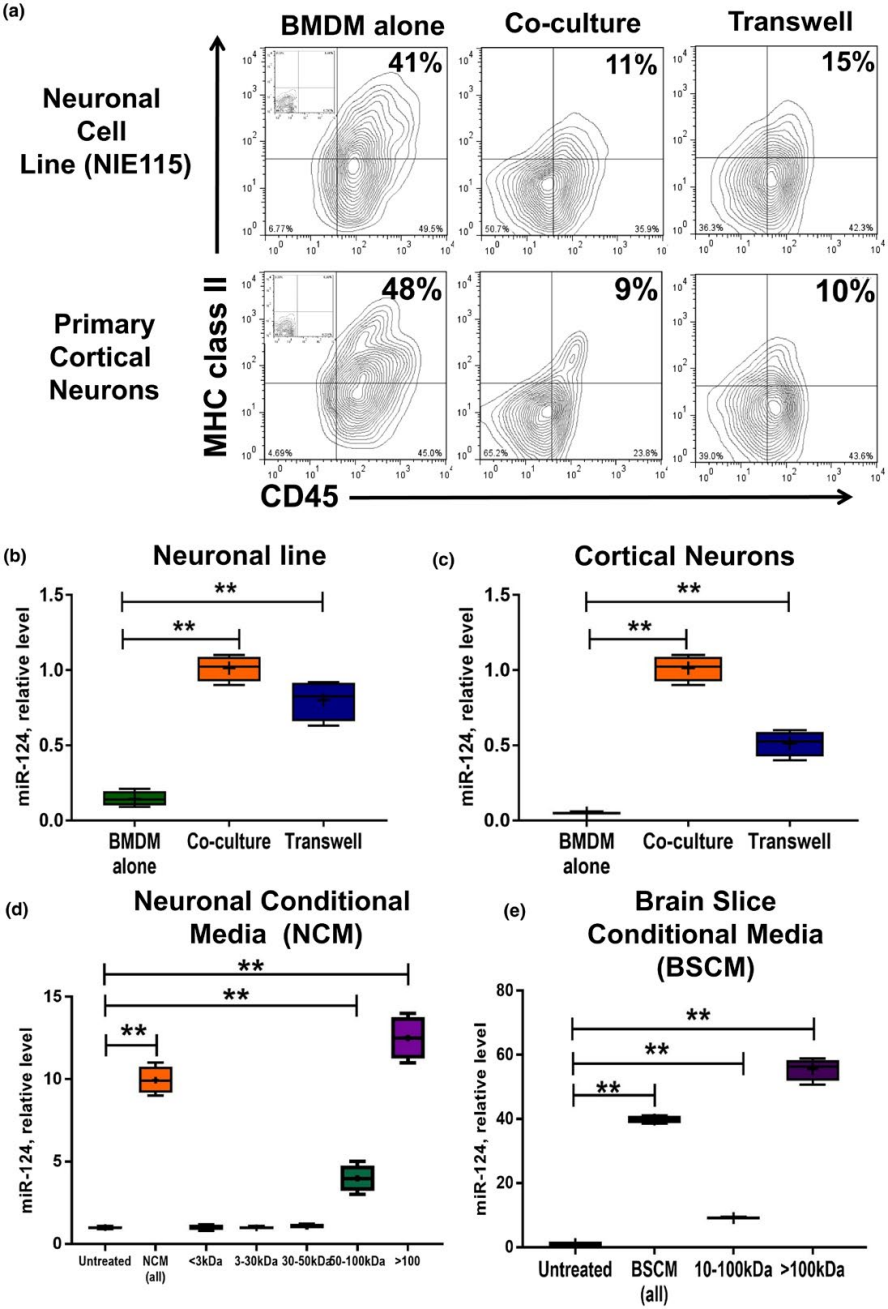
High molecular weight neuronal soluble factors induce downregulation of CD45 and MHC class II and upregulation of miR‐124 in macrophages. (a–c) Bone marrow‐derived macrophages (BMDMs) were obtained from DsRed transgenic mice as described in *Materials and Methods* and cultured alone (BMDMs alone), directly co‐cultured (co‐culture), or co‐cultured separately using a Transwell system (*Transwell*) with neuronal cell line (top contour plots and b) or primary cortical neurons (bottom contour plots and c) for 5 days and analyzed for the expression of DsRed, CD11b, CD45, and MHC class II using 4‐color cytometry. DsRed^+^CD11b^+^ gated macrophages were analyzed for the expression of CD45 (*x*‐axis) and MHC class II (*y*‐axis) (a), or FACS‐sorted DsRed^+^CD11b^+^ macrophages were used for isolation of RNA and analysis of miR‐124 expression by real‐time RT PCR as described in *Materials and Methods* (b, c). (d, e) Fractionation of neuronal conditioned medium (NCM) according to molecular weight and analysis of each fraction for the ability to induce expression of miR‐124 in macrophages. NCM (d) or brain slice conditioned medium (e; BSCM) were prepared and fractionated by size filtration as described in *Materials and Methods*. BMDMs were cultured alone in 50% neurobasal medium (untreated) or cultured in the presence of 50% non‐fractionated NCM (NCM‐all) or BSCM (BSCM‐all) or in fractionated NCM (<3; 3–30; 30–50; 50–100; >100 kDa) or BSCM (10–100; >100 kDa) for 4 hr and analyzed for miR‐124 expression by real‐time RT PCR as described in *Materials and Methods.* In (b–e), the median ± *SD* is shown on box and whisker plots (*n* = 4) with the mean value indicated by “+” symbol. The indicated differences were statistically significant as determined by one‐way ANOVA followed by Bonferroni post hoc test (**p < 0.01 for comparisons between two groups; b: *F*(2, 9) = 86.42, *p* < 0.0001; c: *F*(2, 9) = 190.3, *p* < 0.0001; d: *F*(6, 21) = 216.7, *p* < 0.0001; e: *F*(3, 12) = 775.6, *p* < 0.0001) [Colour figure can be viewed at wileyonlinelibrary.com]

We have previously found that co‐culture of neuronal cell line N1E115 (mouse neuroblastoma) with mouse BMDMs resulted in downregulation of a number of activation markers (MHC class II and CD45) and upregulation of miR‐124 (Ponomarev, Veremeyko, Barteneva et al., 2011). We also found that transfection of BMDMs with miR‐124 resulted in downregulation of MHC class II and CD45 via targeting of CEBPα and PU.1 transcription factors (Ponomarev, Veremeyko, Barteneva et al., 2011). This suggested that neuronal cells could provide direct cell‐contact or soluble‐factor signals to macrophages to cause upregulation of miR‐124 in microglia/macrophages and downregulation of MHC class II and CD45 (Ponomarev et al., 2013). However, it was not determined whether soluble factors from primary neurons could cause a similar effect in terms of deactivation and upregulation of miR‐124. To investigate this, we used co‐cultures of BMDMs with the NE1115 neuronal cell line and with mouse primary cortical neurons. Transwell co‐culture systems were also used to identify the role of neuronal soluble factors in the regulation of expression of MHC class II, CD45, and miR‐124. We found that both neuronal line and primary cortical neurons downregulated MHC class II and CD45 (Figure 1a). Moreover, it was sufficient to have soluble factors to induce MHC class II^low^CD45^low^ phenotype in macrophages (Figure 1a, *Transwell*). Soluble factors from both neuronal cell line (Figure 1b) and primary cortical neurons (Figure 1c) were also sufficient to induce expression of miR‐124 in BMDMs. The quantitative analysis is shown in Table 2. Thus, neuronal soluble factors induced expression of miR‐124 in macrophages, which was associated with their deactivated phenotype.

**Table 2:** Quantitative analysis of the expression of activation markers MHC class II and CD45 on macrophages cultured alone, directly co‐cultured with neuronal cells, or cultured with neuronal cells using transwell system†

Neuronal cells	BMDM alone (no co‐culture)	Direct co‐culture with neuronal cells	Co‐culture with neuronal cells using a transwell system
Neuronal	42 ± 10	11 ± 3**	16 ± 3**
Cell			
Line (N1E115)			
Primary		9 ± 2**	10 ± 2**
Cortical			
Neurons			

^†^ Bone‐marrow‐derived macrophages (BMDM) were cultured alone (no co‐culture), directly co‐cultured with neuronal cell line N1E115 and primary cortical neurons, or co‐cultured with neuronal cells using a transwell system as for Figure 1a and the percentages of MHC class II^+^CD45^+^ double‐positive cells is shown. Mean ±* S* *D* of separate experiments is shown (*n* = 3).

** The difference is statistically significant when compared to BMDM cultured alone as determined by one‐way ANOVA (*F*(2,10) = 24,7; *p* < 0.0001) followed by Bonferroni post hoc test (*p* < 0.01 for single comparisons between two groups).

### 3.2 High molecular weight fractions of 50–100 and >100 kDa of neuronal conditioned media induced miR‐124 in macrophages

To further characterize neuronal soluble factors, we used neuronal conditioned media (NCM), which was added (1:1) to BMDMs. As an untreated control, we added (1:1) fresh medium when primary neurons or neuronal line were incubated. We found that NCM upregulated miR‐124 in macrophages. We investigated whether NCM specifically induces miR‐124 in BMDMs or whether other abundant neuronal microRNAs such as miR‐9 could also be induced. We found that incubation of BMDMs with NCM resulted in 15.7‐fold upregulation of miR‐9 (not shown). Next, we performed fractionation of NCM and found that the most active fraction for miR‐124 induction was a high molecular weight fraction of >100 kDa followed by a smaller fraction of 50–100 kDa (Figure 1d). We have previously found that IL‐4 and IL‐13 (but neither IL‐10 nor TGFβ1) upregulated miR‐124 in BMDMs, whereas IL‐10 and TGFβ1 deactivated macrophages. Because most of the regulatory cytokines that deactivate macrophages and/or induce miR‐124 (e.g., IL‐4, IL‐13, IL‐10, TGFβ), growth factors, and nutrients have the relatively low molecular weight of <50 kDa, we hypothesized that somewhat larger molecular complexes sized at >50 kDa mediated expression of miR‐124 in macrophages. As an alternative to NCM, we used BSCM that was obtained from neuronal organotypic cultures. We found that, similar to NCM, BSCM also induced miR‐124 but to a higher degree—not surprising given the large numbers of both neuronal and glial cells in acute brain slice organotypic cultures (Figure 1e). Similar to NCM, a fraction of >100 kDa was the most effective in induction of miR‐124 in macrophages (Figure 1e). Taken together, these data indicate an important role of high molecular weight complexes in the induction of miR‐124 in macrophages. We also found that NCM induced expression of another neuronal microRNA (miR‐9) in macrophages.

### 3.3 Microglia express miR‐124 and miR‐9 only in the CNS microenvironment, whereas both bone marrow‐ and yolk sac‐derived macrophages upregulated miR‐124 after incubation with neuronal conditioned media

**Figure 2:**
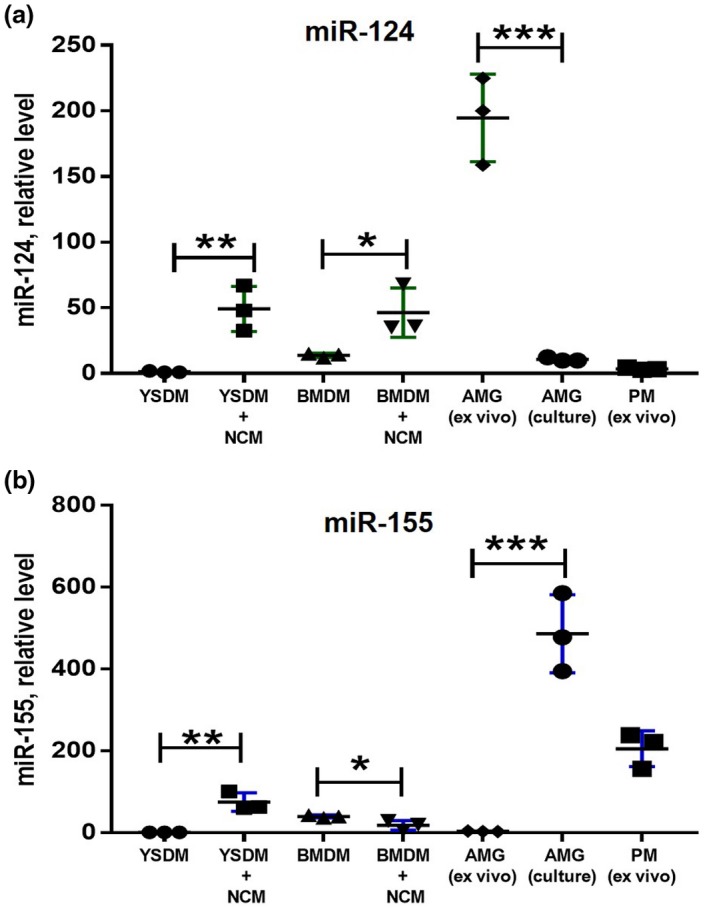
Comparison of expression of miR‐124 and miR‐155 in bone marrow‐ and yolk sac‐derived macrophages under the influence of neuronal conditioned media. Bone marrow‐derived macrophages (BMDMs), yolk sac‐derived macrophages (YSDMs), or adult microglia were grown in the presence of M‐CSF as described in *Materials and Methods*. BMDMs and YSDMs were incubated in 50% neurobasal media (YSDM and BMDM) or 50% NCM (YSDM + NCM and BMDM + NCM) for 4 hr, and the expression levels of miR‐124 (a) and miR‐155 (b) were analyzed by real‐time RT PCR as described in *Materials and Methods*. The level of expression of miR‐124 and miR‐155 in YSDMs and BMDMs was compared to ex‐vivo isolated (AGM (ex vivo)) and cultured (AGM(culture)) adult microglia and ex‐vivo isolated peritoneal macrophages (PM (ex vivo)). In (a, b), mean ± *SD* of three separate experiments is shown on dotplots (*n* = 3; *p < 0.5; **p < 0.01; ***p < 0.001 as determined by unpaired Student's *t* test) [Colour figure can be viewed at wileyonlinelibrary.com]

In our experiments, we used BMDMs to induce miR‐124 under the influence of NCM (Figure 1). However, it was established that microglia originate from yolk sac (YS) but not from bone marrow (BM) (Ponomarev et al., 2013). Therefore, YS‐derived macrophages may react differently to NCM. Moreover, YS‐derived macrophages and adult microglia may have an intrinsic level of miR‐124 expression. To answer this question, we compared the level of miR‐124 expression in BM‐ and YS‐derived macrophages grown in M‐CSF and in ex vivo isolated and cultured adult microglia. We also measured the miR‐124 expression level in resident peritoneal macrophages, which are known to originate from BM. We found that cultured YS‐derived macrophages had an even lower level of expression of miR‐124 in comparison to BMDMs (Figure 2a, *YSM *vs. *BMM*). NCM induced miR‐124 in both BM‐ and YS‐derived macrophages to similar levels (Figure 2a, *YSM + NCM *vs. *BMM + NCM*). Ex vivo isolated adult microglia, but not peritoneal macrophages, had the highest level of miR‐124 (Figure 2a, *AMG *(ex vivo) versus *PM *(ex vivo)). However, when adult microglia were cultured in the presence of M‐CSF for several weeks, these cells dramatically decreased their level of miR‐124 to the level of BM‐derived macrophages (Figure 1a, *AMG (culture)*). Ex vivo isolated microglia also expressed miR‐9, which was not detected in cultured adult microglia (not shown). We also investigated the expression of miR‐155, which was upregulated in classically activated M1 macrophages and often opposes the pattern of expression of miR‐124, which is expressed more in alternatively activated M2 macrophages (Ponomarev et al., 2013; Veremeyko et al., 2013, 2018 ). We found that NCM induced miR‐155 in YS‐, but not in BM‐derived macrophages. MiR‐155 was also upregulated in cultured adult microglia, and this microRNA was also expressed on peritoneal macrophages (Figure 2b). Thus, we found that neuronal soluble factors induced miR‐124 in both BM‐ and YS‐derived macrophages and that adult microglia expressed miR‐124 only in the context of the CNS microenvironment.

### 3.4 Various protein:RNA complexes are present in the fraction of 30–100 kDa that are sensitive to RNase treatment

**Figure 3:**
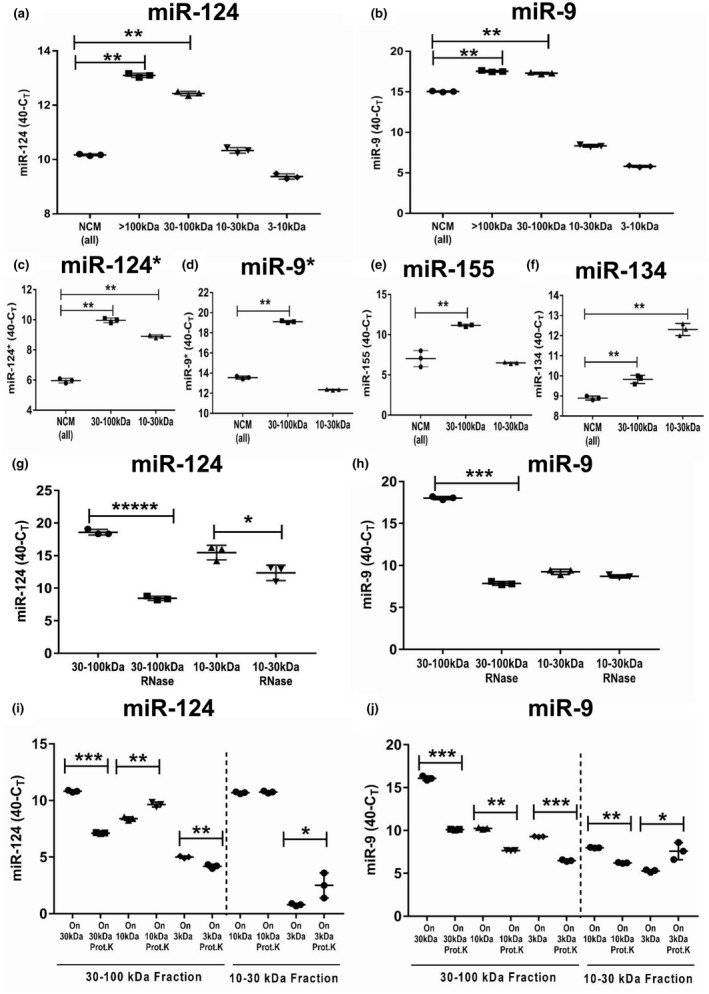
Analysis of the level of expression, RNase sensitivity, and adsorption to proteins of neuronal microRNAs miR‐124, miR‐9, miR‐124*, miR‐9*, miR‐155, and miR‐134 in various fractions of neuronal conditioned medium (NCM). NCM was harvested from cultured neurons, fractionated by size filtration, and then further used as untreated (a–f) or treated with RNase (g, h) or with proteinase (i, j), and analyzed for the expression of neuronal miRNAs as described in *Materials and Methods*. (a, b) Analysis of expression of miR‐124 (a) and miR‐9 (b) in unfractionated NCM (NCM‐all) and in a fraction of >100 kDa (>100 kDa), and fractions of 30–100, 10–30, and 3–10 kDa. (c–f) Analysis of the expression of miR‐124* (c), miR‐9* (d), miR‐155 (e), and miR‐134 (f) in unfractionated NCM (NCM‐all) in fractions of 30–100 and 10–30 kDa. (g) Comparison of expression of miR‐124 in untreated NCM versus NCM treated with RNase in fractions of 30–100 and 10–30 kDa. (h) Comparison of expression of miR‐9 in untreated NCM versus NCM treated with RNase in fractions of 30–100 and 10–30 kDa. (i) Comparison of expression of miR‐124 in untreated NCM versus NCM‐treated proteinase K in fractions of 30–100 and 10–30 kDa. The 30–100‐kDa fraction (left) was used as untreated or treated with proteinase K and then passed through 30‐, 10‐, and 3‐kDa filters. The fractions that remained on the 30‐, 10‐, and 3‐kDa filters were analyzed for the expression of miR‐124. The 10–30‐kDa fraction (right) was used as untreated or treated with proteinase K and then passed through 10‐ and 3‐kDa filters. The fractions that remained on the 10‐ and 3‐kDa filters were analyzed for the expression of miR‐124. (j) Comparison of the expression of miR‐9 in untreated NCM versus NCM treated with proteinase K in fractions of 30–100 and 10–30 kDa. The 30–100‐kDa fraction (left) was used as untreated or treated with proteinase K and passed through 30‐, 10‐, and 3‐kDa filters. The fractions that remained on the 30‐, 10‐, and 3‐kDa filters were analyzed for the expression of miR‐9. The 10–30‐kDa fraction (right) was used as untreated or treated with proteinase K and then passed through 10‐ and 3‐kDa filters. The fractions that remained on the 10‐ and 3‐kDa filters were analyzed for the expression of miR‐9. In (a–j), mean ± *SD* of three separate experiments is shown on dotplots (*n* = 3). In (a–f), the indicated differences were statistically significant as determined by one‐way ANOVA followed by Bonferroni post hoc test (**p < 0.01 for comparisons between two groups; a: *F*(4, 10) = 1,177, *p* < 0.0001; b: *F*(4, 10) = 7,154, *p* < 0.0001; c: *F*(2, 6) = 688.4, *p* < 0.0001; d: *F*(2, 6) = 3,243, *p* < 0.0001; e: *F*(2, 6) = 56.93, *p* = 0.0001; f: *F*(2, 6) = 196.9, *p* < 0.0001). In (g–j), the indicated differences were statistically significant as determined by unpaired Student's *t* test (*p < 0.5; **p < 0.01; ***p < 0.001; *****p < 0.00001) [Colour figure can be viewed at wileyonlinelibrary.com]

Next, we investigated the presence of miR‐124 in various fractions of NCM. As an additional neuronal miRNA that was upregulated in macrophages by NCM, we investigated miR‐9. The highest concentrations of both miR‐124 (Figure 3a) and miR‐9 (Figure 3b) were found in the >100‐kDa fraction. However, lower molecular weight fractions (30–100, 10–30, and 3–10 kDa) contained both miR‐124 and miR‐9 (Figure 3a,b). Fractions 30–100 and 10–30 kDa also contained many other types of neuronal miRNAs besides miR‐124 and miR‐9: miR‐124* (Figure 3c), miR‐9* (Figure 3d), miR‐155 (Figure 3e) and miR‐134 (Figure 3f). We investigated whether fractions 30–100 and 10–30 kDa were sensitive to RNase treatment. We found that the fraction of 30–100 kDa was sensitive to RNase treatment, leading to a 2‐ to 3‐fold decrease in miR‐124 (Figure 3g) and miR‐9 (Figure 3h) levels in the 30–100‐kDa fraction. Of interest, the 10–30‐kDa fraction was less sensitive to RNase treatment. This indicates that there are smaller proteins of specific conformation and/or double‐stranded RNA formations that block RNase from digesting these miRNAs.

To demonstrate that proteins do form complexes with miR‐124 or miR‐9, we enzymatically treated 30–100‐kDa complexes with proteinase K and then passed them again through the same size 30‐kDa filter and smaller size (10 and 3 kDa) filters. Similar treatments were performed for 10–30‐kDa fraction, which we then passed through the 10‐ and 3‐kDa filters. We found that the amounts of miR‐124 (Figure 3i) and miR‐9 (Figure 3j) in the 30–100‐kDa fraction were significantly decreased on the 30‐kDa filter when these fractions were treated with proteinase K and then passed through the same filter when compared to the untreated control (Figure 3i,j; *On 30 kDa*). For miR‐124, the amount of this miRNA was increased on the 10‐kDa filter when the 30–100‐kDa fraction was treated with proteinase K and then passed through the 10‐kDa filter. This indicates the passage of deproteinized miR‐124 through the 30‐kDa filter and retention of deproteinized miR‐124 on the 10‐kDa filter (Figure 3i,j; *On 10 kDa*). At the same time, proteinase treatment of the 30–10‐kDa fraction did not significantly affect the amount of miR‐124 retained on the 10‐kDa filter (Figure 3i; *On 10 kDa*), indicating the presence of miR‐124 in the naked form in this fraction. In contrast to miR‐124, proteinase treatment of the 30–100 and 10–30‐kDa fractions decreased the amount of miR‐9 in all filters except for the 3‐kDa filter (Figure 3j; *On 3 kDa*). These results suggest that miR‐9 was probably present in single‐chain form of around 7 kDa that passed through the 10‐kDa filter but was retained on the 3‐kDa filter. At the same time, miR‐124 probably formed miR‐124:miR‐124* duplexes with molecular weight around 14 kDa, which is higher than 10 kDa, explaining the retention of deproteinized miR‐124 on the 10‐kDa filter. An increase in the amount of proteinase K‐treated miR‐124 and miR‐9 on the 3‐kDa filter indicates that dissociation of microRNAs from proteins facilitated their passage through the 30 and 10‐kDa filter and retention of naked RNA on 3‐kDa filter. Thus, our data indicate that the fraction of 30–100 kDa contains multiple protein complexes with miR‐124 and miR‐9, whereas the smaller fractions (10–30 kDa) contain deproteinized (naked) RNAs.

### 3.5 The fraction of >100 kDa contains both protein:miR‐124 and protein:miR‐9 complexes and exosomes with miR‐9 and miR‐155

**Figure 4:**
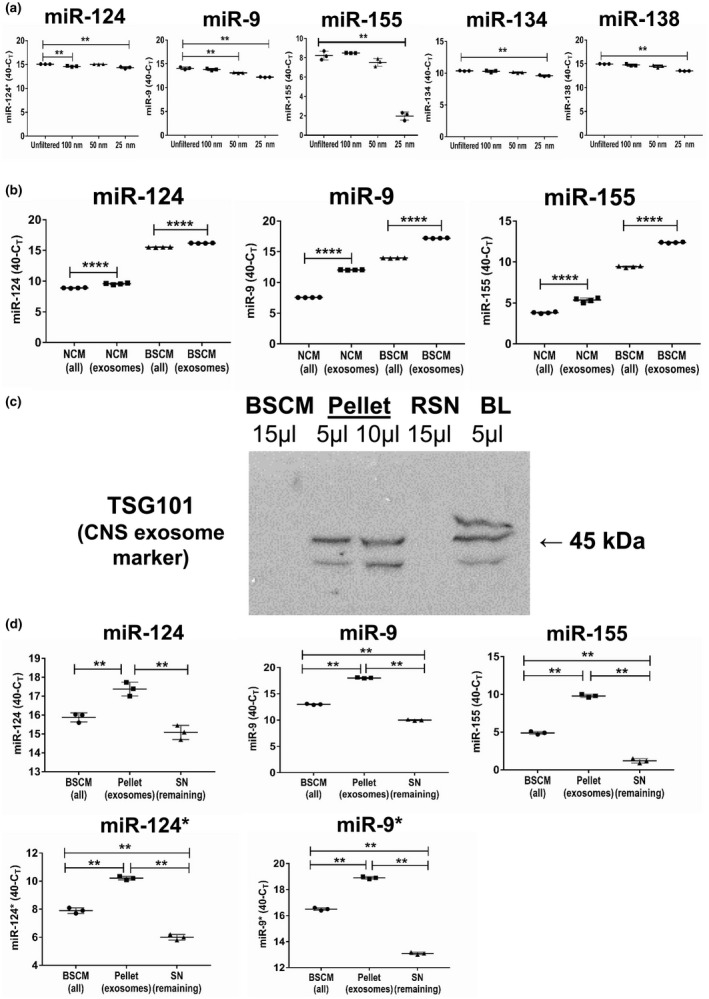
Analysis of the level of expression of neuronal microRNAs miR‐124, miR‐9, miR‐155, MiR‐134, and miR‐138 in microparticles and exosomes. (a) Neuronal conditioned medium (NCM) was harvested from cultured neurons as described in *Materials and Methods* and used unfiltered or passed through 100‐, 50‐ and 25‐nm filters and analyzed for the expression of miR‐124, miR‐9, miR‐155, miR‐134, and miR‐138. (b) NCM or brain slice conditioned medium (BSCM) were harvested from cultured neurons or organotypic brain slices, and exosomes were prepared as described in *Materials and Methods*. The levels of expression of miR‐124, miR‐9 and miR‐155 were measures for whole NCN (NCM‐all) or whole BSCM (BSCM‐all) versus exosomal fraction isolated from NCM (NCM‐exosomes) or BSCM (BSCM‐exosomes). (c) Western blot analysis of the expression of CNS exosomal marker TSG101 in the whole BSCM (15 μl), pellet (exosome preparation; 5 and 10 μl), and remaining supernatant (RSN, 15 μl). Brain lysate was used as a positive control (BL, 5 μl). Exosomes were precipitated from 10 ml of BSCM using total exosome isolation reagent (from cell culture medium) as described in *Materials and Methods*. Pellet with exosomes was re‐suspended in 100 μl of Laemmli lysis buffer for electrophoresis and further western blot analysis. (d) Comparison of the level of expression of miR‐124, miR‐9, miR‐155, miR‐124*, and miR‐9* in the whole BSCM (BSCM‐all), pellet (exosomes), and remaining supernatant (Remaining SN). In (a, d), the mean ± *SD* of triplicate is shown on dotplot graphs (*n* = 3). The indicated differences were statistically significant as determined by one‐way ANOVA followed by Bonferroni post hoc test (**p < 0.01 for comparisons between two groups; a, miR‐124: *F*(3, 8) = 27.96, *p* = 0.0001; a, miR‐9: *F*(3, 8) = 72.09, *p* < 0.0001; a, miR‐155: *F*(3, 8) = 208.1, *p* < 0.0001; a, miR‐134: *F*(3, 8) = 27.32, *p* = 0.0001; a, miR‐138: *F*(3, 8) = 44.65, *p* < 0.0001; d, miR‐124: *F*(2, 6) = 36.76, *p* = 0.0004; d, miR‐9: *F*(2, 6) = 5,018, *p* < 0.0001; d, miR‐155: *F*(2, 6) = 1,002, *p* < 0.0001; d, miR‐124*: *F*(2, 6) = 418.8, *p* < 0.0001; d, miR‐9*: *F*(2, 6) = 2,548, *p* < 0.0001). In (b), mean ±* SD* of quadruplicate is shown on dotplot graphs (*n* = 4; ****, *p* < 0.0001; unpaired Student's *t* test) [Colour figure can be viewed at wileyonlinelibrary.com]

We further investigated the role of the >100‐kDa fraction, as this fraction demonstrated the most dramatic biological effect in the induction of miR‐124 in BMDMs (Figure 1d). A large fraction of more than 100 kDa may contain large protein:RNA complexes and microparticles of various sizes that include microparticles (100–1,000 nm) and exosomes (30–90 nm). Because it is known that exosomes and larger microparticles contained miRNA, we performed filtration of NCM through 100‐, 50‐, and 25‐nm nylon filters. We found that only the 50‐ and 25‐nm filters decreased the level of miR‐155 and, to a lesser degree, miR‐9, but not miR‐124, miR‐134, and miR‐138 (Figure 4a). These data indicate that NCM contain small exosomes with miR‐155 and miR‐9, whereas other neuronal microRNAs (e.g., miR‐124) were found in protein:RNA complexes. To verify that miR‐155 and miR‐9 but not miR‐124 were found in exosomes, we selectively isolated exosomes from both NCM and BSCM using a kit from Invitrogen (see *Materials and Methods*) and found that miR‐9 and miR‐155 were enriched in the precipitated exosome fraction (Figure 4b). Thus, these data indicate that for the most of the studied microRNAs only proportion of them was enriched in exosomal fraction, with the exception of miR‐155. For miR‐155, about 80% of this miRNA was retained by 25 nm filter that eliminate exosomes, while for miR‐9 and miR‐124 these numbers were ~15% and ~5%, respectively (Figure 4a).

We further confirmed that the precipitated (pellet) fraction of exosomes expressed a high level of the neuronal exosome marker TSG101, which was also present in brain lysate (Figure 4c, *Pellet *and *BL*), whereas this exosomal marker was not detected in whole BSCM or in the non‐precipitated fraction of remaining supernatant (Figure 4c, *BSCM* and *RSN*). Further analysis demonstrated that miR‐155 and miR‐9, and, to a lesser degree, miR‐124* and miR‐9* were enriched in the exosomal fraction when compared to the remaining supernatant, which was not the case for miR‐124 (Figure 4d). It is quite clear from the filtration and exosome enrichment experiments that the size of exosomes in the conditioned media was in the range from 25 to 100 nm (Figure 4a; *miR‐155*; Figure 4d, *miR‐155*). Thus, miR‐155 was mainly co‐localized with exosomes; miR‐9, miR‐9*, and miR‐124* were found in both exosomes and remaining supernatant, whereas miR‐124 was predominantly found in the remaining supernatant.

### 3.6 MiR‐124 and miR‐9 form complexes with HDLs

**Figure 5:**
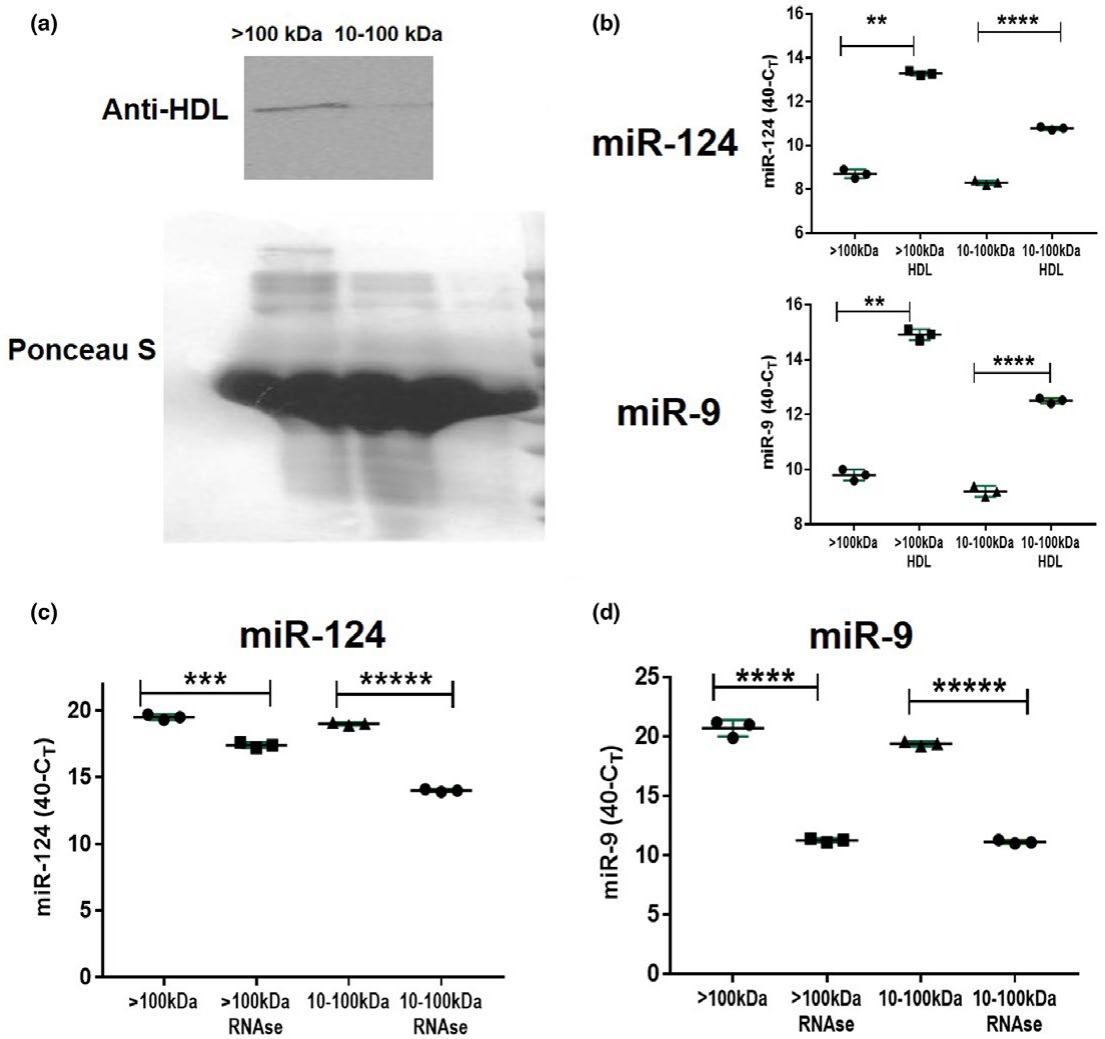
Analysis of the level of expression of neuronal microRNAs miR‐124 and miR‐9 in HDL complexes. (a) Western blot analysis of the expression of 40‐kDa protein from HDL complexes in high molecular weight (>100 kDa) versus low molecular weight (10–100 kDa) fractions of BSCM. Ponseus S staining was used as a protein loading control. (b) Immunoprecipitation of HDL with miR‐124 and miR‐9. Anti‐HDL antibodies were adsorbed to plastic and >100‐kDa or 10–100‐kDa fractions of BSCM were added to antibodies as described in *Materials and Methods*. Expression of miR‐124 was assessed in HDLs bound to anti‐HDL antibodies (>100 kDa:HDL and 10–100 kDa:HDL) and in solution unbound to HDL in the >100 and 10–100‐kDa fractions (>100 and 10–100 kDa). (c, d) Comparison of expression of miR‐124 (c) miR‐9 (d) in untreated BSCM versus BSCM treated with RNase (fractions of >100 and 10–100 kDa). In (b–d), mean ±* SD* of three separate experiments is shown on dotplots (*n* = 3; **p < 0.01; ***p < 0.001; ****p < 0.0001; ****p < 0.00001; unpaired Student's *t* test) [Colour figure can be viewed at wileyonlinelibrary.com]

Previous experiments led us to hypothesize that miR‐124 is probably located in large protein:RNA complexes in NCM or BSCM. One of the known large protein complexes of the size >100 kDa are HDLs. HDLs are known to be present in the CNS, where they are produced by astrocytes and microglia (Vitali et al., 2014). It was reported that microRNA has the ability to bind to lipids within HDL complexes, which have the ability to translocate into the cytoplasm of recipient cells (Vickers et al., 2011). We looked at the fractions of BSCM of >100 and 10–100 kDa by western blot using antibodies that recognize 40‐kDa protein from HDL complexes. We found that HDLs were mostly present in the >100‐kDa fraction of BSCM and, to a much lesser degree, in the 10–100‐kDa fraction (Figure 5a). Similar results were found for NCM (not shown). Immunoprecipitation experiments further confirmed that miR‐124 and miR‐9 were in complex with HDLs that were abundant in the >100‐kDa fraction (Figure 5b). When we treated a fraction of >100 kDa with RNase, we found that miR‐124 was resistant to RNase in this fraction (Figure 5c). At the same time, miR‐9 was slightly more sensitive to RNase treatment in this fraction but still quite resistant (Figure 5d). These results suggest that miRNA:HDL complexes were mostly resistant to RNase treatment. Thus, most biologically active fractions of >100 kDa contained miR‐124:HDL complexes resistant to RNase.

### 3.7 MiR‐124 and miR‐9 are promptly released from neuronal cells and gradually accumulated in the media

**Figure 6:**
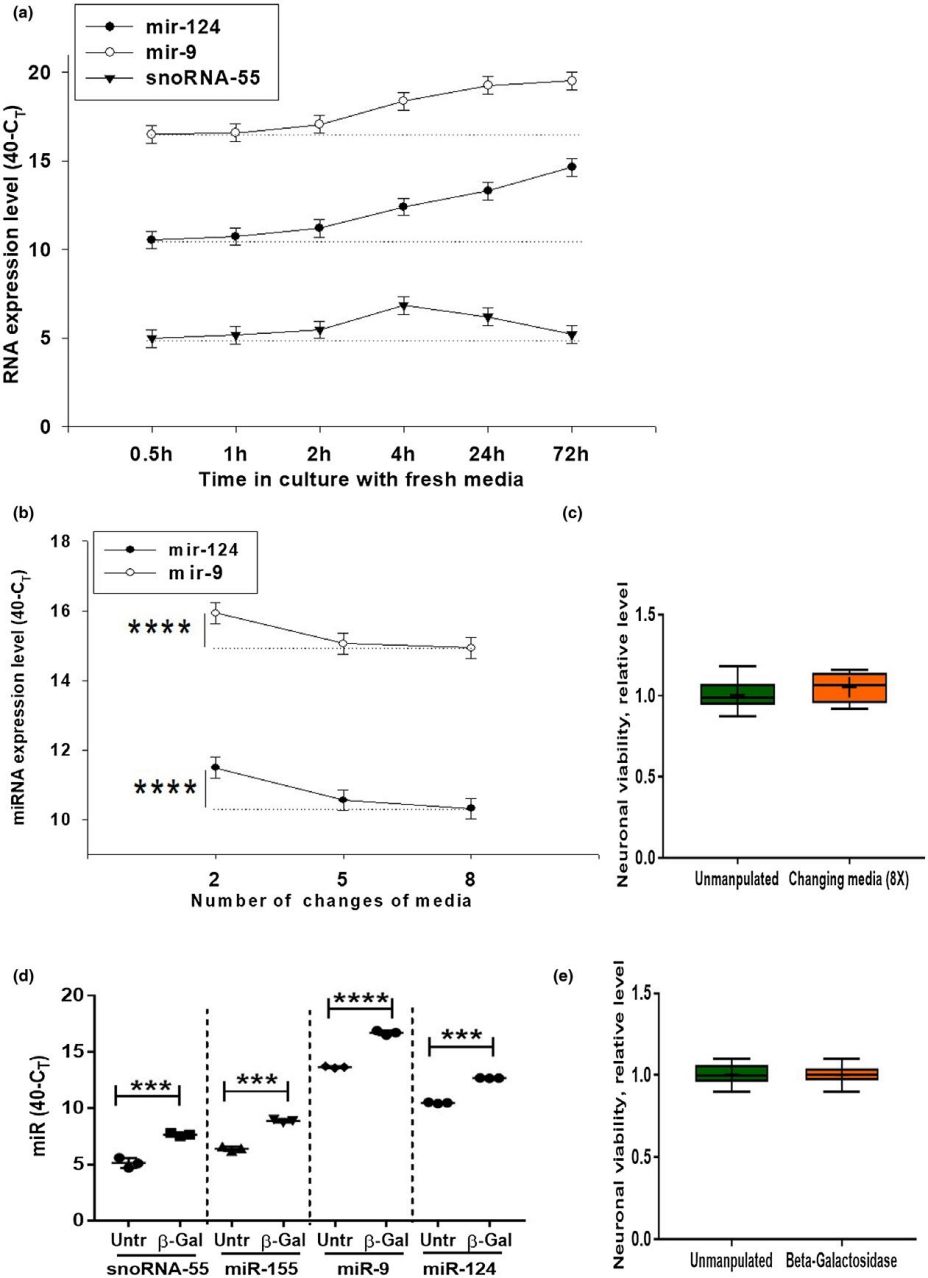
Kinetics of extracellular production of miR‐124 and miR‐9 by cultured cortical neurons. (a) Primary mouse cortical neurons were cultured as described in *Material and Methods*. The medium was changed once at 0 hr time point, and the expression of miR‐124, miR‐9, and snoRNA‐55 were assessed 0.5, 1, 2, 4, 24, and 72 hr later by real‐time RT PCR as described in *Materials and Methods. *(b, c) Primary mouse cortical neurons were cultured as described in *Material and Methods*. The medium was changed 2 or 5 or 8 times, and expression of miR‐124 and miR‐9 (b) or neuronal viability (c) were assessed by real‐time RT PCR or bioluminescent viability kit, respectively, as described in *Materials and Methods. *(d, e) Primary mouse cortical neurons were used untreated and treated with β‐galactosidase for 30 min (d, e), and the expression levels of miR‐124 and miR‐9 (d) or neuronal cell viability (e) were assessed by real‐time RT PCR or bioluminescent viability kit, respectively, as described in *Materials and Methods. *In (a, b), mean ±* SD* of five separate culture wells is shown. In (b), ****p < 0.0001, as determined by repeated measures ANOVA test (miR‐124: *F*(2,12) = 84.5; miR‐9: *F*(2,12) = 45.2). In (d), mean ±* SD* of three separate cell culture wells is shown (***p < 0.001; ****p < 0.0001 when compared to the same sample before the treatment with β‐galactosidase; paired Student's *t* test). In (c, e), mean ±* SD* of six separate cell culture wells is shown on box and whisker plot with the mean value indicated by “+” symbol [Colour figure can be viewed at wileyonlinelibrary.com]

After characterization of various fractions of NCM and BSCM, we investigated the kinetics of how miR‐124 and miR‐9 were produced by cultured cortical neurons. As a control, we used small snoRNA‐55, which is abundantly expressed in neuronal cells and is often used as a housekeeping RNA for normalization of expression of miRNA. Surprisingly, we found quite a high level of expression of miR‐9 and miR‐124 as early as 30 min of incubation of primary neurons in fresh neurobasal medium. At the same time, snoRNA‐55 was found in quite a low level (Figure 6a; *0.5 hr*). We checked the levels of expression of miR‐124, miR‐9, and snoRNA‐55 in fresh neurobasal medium and could not detect these molecules after 40 cycles of amplification (not shown). Thus, these molecules appeared to be promptly released by cultured neuronal cells. The amounts of miR‐9 and miR‐124 were then gradually increasing during the 72‐hr period (Figure 6a; *miR‐124* and *miR‐9*). In contrast to miR‐124 and miR‐9, the level of snoRNA‐55 was increased after 4 hr of incubation and then started to decline to reach a level at 72 hr similar to what it was after 1 hr of incubation (Figure 6a; *snoRNA‐55*). Thus, we concluded that miR‐124 and miR‐9 were not passively released from dead cells and accumulated in a nonspecific way as did the other short RNAs such as snoRNA‐55. Our data indicated a distinct pathway for secretion of neuronal microRNAs by cultured neurons.

### 3.8 MiR‐124 and miR‐9 are accumulated on the surface of neuronal cells

We hypothesized that miR‐124 and miR‐9 were detected in high concentrations in media just after 30 min of incubation as a result of large depositions of these microRNAs on the cell surface. Several studies suggested that miRNA could bind lipids (Khvorova et al., 1999), so we performed detection of miR‐124 and miR‐9 after 2, 5, and 8 changes of medium. Each time, cells were incubated in fresh medium for 1 min and before media were changed or collected for analysis of miR‐124 or miR‐9. Indeed, after 5 or 8 changes of medium, the concentrations of miR‐124 and miR‐9 were significantly decreased (Figure 6b). At the same time, 8 times changing of medium did not decrease neuronal viability (Figure 6c). This indicated that these microRNAs were washed out from cells. To further investigate whether miR‐124 and miR‐9 were bound to the cell surface, we treated neuronal cells with β‐galactosidase to remove carbohydrates from cell surface glycolipids and glycoproteins. Treatment of cells with β‐galactosidase for 30 min increased concentrations of snoRNA‐55, miR‐155, miR‐9, and miR‐124 in NCM (Figure 6d) and did not change neuronal viability (Figure 6e). Thus, miRNAs were present on the surface of cells, resulting in large depositions of these molecules on plasma membrane binding to glycolipids and glycoproteins.

### 3.9 Monensin and ionomycin modulate the release of miR‐124 and miR‐9 by neuronal cells

**Figure 7:**
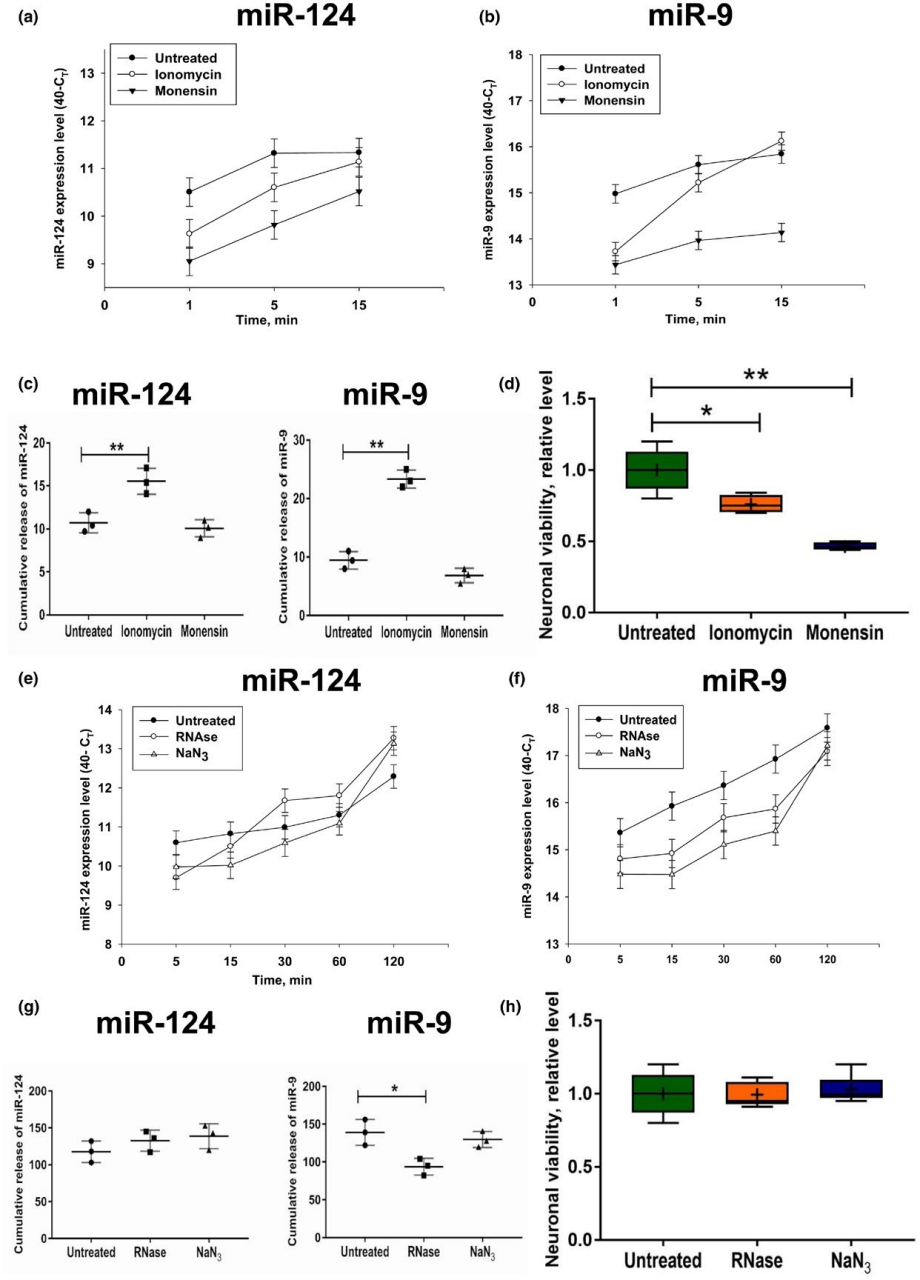
Influence of monensin, ionomycin, sodium azide, and RNase treatment on the level of extracellular production of miR‐124 and miR‐9 by cultured cortical neurons. (a–d) Primary mouse cortical neurons were cultured as described in *Materials and Methods.* The cells were untreated or pre‐treated with ionomycin or monensin for 30 min, the media were changed to fresh media without or with ionomycin or monensin at the 0‐min time point, and the expression levels of miR‐124 (a), and miR‐9 (b) were assessed 1, 5, and 15 min later by real‐time RT PCR as described in *Materials and Methods. *Cumulative values of miRNA release were calculated as area under curves for miRNA release from the 1 to 15 min period (see *Materials and Methods*) (c). Neuronal viability is shown in (d). (e–h) Primary mouse cortical neurons were cultured as described in *Materials and Methods.* The cells were untreated or pre‐treated with RNase or sodium azide for 30 min, the media was changed to fresh media without or with RNase or sodium azide at the 0‐min time point. The expression levels of miR‐124 (e) and miR‐9 (f) were assessed 5, 15, 30, 60, and 120 min later by real‐time RT PCR as described in *Materials and Methods. *Cumulative values of miRNA release (see *Materials and Methods*) are shown in (g), and neuronal viability is shown in (h). In (a–c, e–g), the mean ±* SD* of three separate experiments is shown (*n* = 3). In (d, h), the mean ±* SD* of 4–6 separate cell culture wells is shown on box and whisker plot with the mean value indicated by “+” symbol (*n* = 4–6). The indicated differences were statistically significant as determined by one‐way ANOVA followed by Bonferroni post hoc test (*p < 0.05 and **p < 0.01 for comparisons between two groups; c, miR‐124: *F*(2, 6) = 17.24, *p* = 0.0033; c, miR‐9: *F*(2, 6) = 114.6, *p* < 0.0001; d: *F*(2,10) = 31.4, *p* < 0.0001; g, miR‐9: *F*(2, 6) = 9.849, *p* = 0.0127) [Colour figure can be viewed at wileyonlinelibrary.com]

It was reported that exosomes and HDL:miRNA complexes were released through exocytosis (Vickers et al., 2011). To test whether this mechanism takes place in case of secretion of miR‐124 and miR‐9, we pre‐treated cells with the exocytosis inhibitor monensin or the exocytosis stimulator ionomycin for 30 min, changed media, and investigated the accumulation of miR‐124 and miR‐9 in media within a 15‐min period. Before the treatment, the expression of miR‐124 was the same across culture plates (not shown). Both monensin and ionomycin decreased the levels of miR‐124 (Figure 7a) and miR‐9 (Figure 7b) when measured 1 min after changing of media. We found that monensin did not significantly decrease the rate of accumulation of miR‐124 or miR‐9, whereas ionomycin significantly enhanced it as determined by cumulative release during the 14‐min period from 1 to 15 min of incubation (Figure 7c). At the same time, we found that ionomycin decreased neuronal viability by ~20%, whereas monensin decreased it by ~50% (Figure 7d). There is a possibility that dying neurons passively release miRNAs when treated with ionomycin. However, this was not likely, in that a more dramatic decrease in neuronal viability by monensin (Figure 7d, *Monensin*) did not significantly change miR‐124 and miR‐9 release (Figure 7c, *Monensin*). This result indicated that calcium influx induced by ionomycin stimulated secretion of miR‐124 and miR‐9.

### 3.10 RNase and sodium azide treatments delay the release of miR‐124 and miR‐9 by neuronal cells

It was reported that most of the miRNA:protein complexes were protected from RNase treatment, whereas inhibition of mitochondria functions blocked the secretion of several types of ECmiRNAs (Wang, Zhang, Weber, Baxter, & Galas, 2010). We also investigated whether RNase or the mitochondria inhibitor NaN_3_ affects secretion of miR‐124 and miR‐9. We found that both agents decreased the rate of release of miR‐124 and miR‐9 within a 5–15‐min period but had little effect in a long‐term perspective (15–120 min) (Figure 7e,f). Sodium azide did not significantly affect the cumulative release value for miR‐124 and miR‐9 during the 115‐min period from 5 to 120 min of incubation (Figure 7g). RNase did not affect the cumulative release value for miR‐124 but decreased the cumulative release of miR‐9 (Figure 7g). Neither RNase nor NaN_3 _decreased neuronal viability (Figure 7h). These data indicate that inhibition of mitochondria or RNase treatments delayed the release of miR‐124 and/or miR‐9 in the extracellular space but did not significantly affect this process in the long‐term perspective.

### 3.11 Tetrodotoxin inhibits prompt release of miR‐124 and miR‐9 by neuronal cells

**Figure 8:**
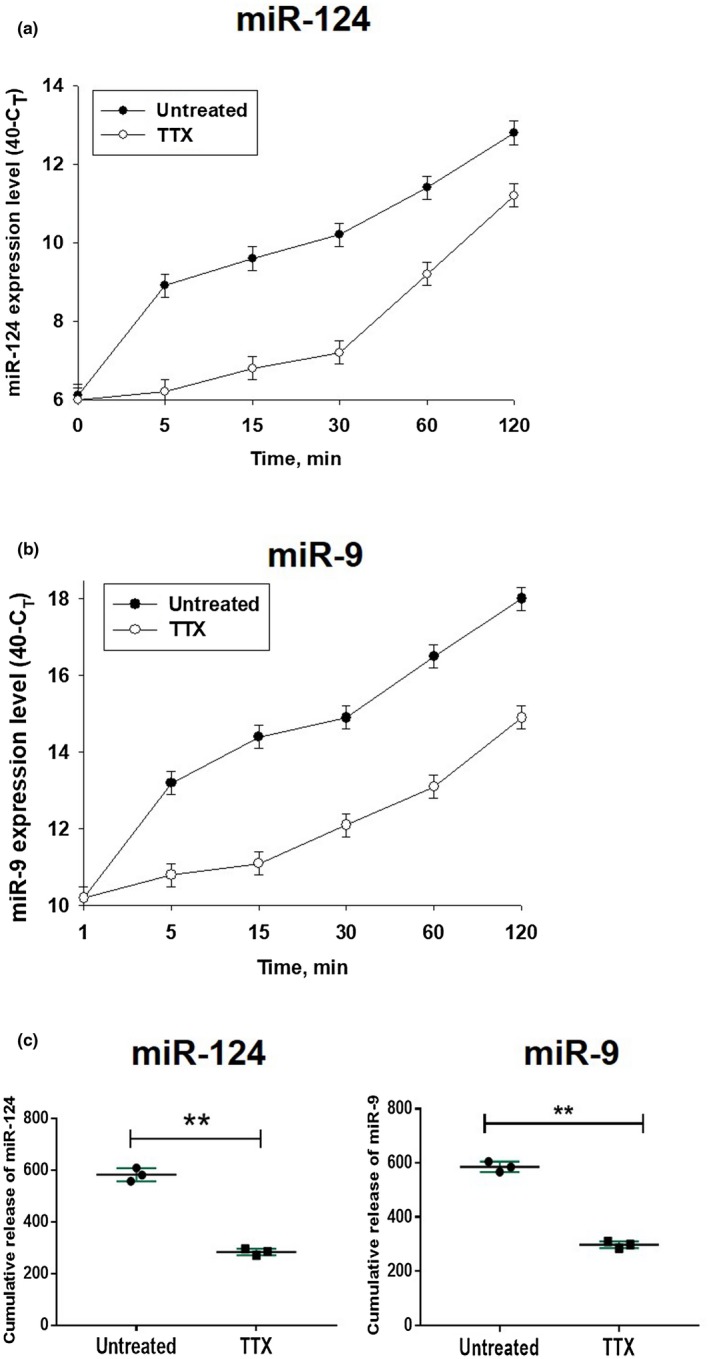
Influence of tetrodotoxin on the level of extracellular production of miR‐124 and miR‐9 by cultured cortical neurons. Primary mouse cortical neurons were cultured as described in *Materials and Methods.* The cells were untreated or pre‐treated with tetrodotoxin (TTX) for 24 hr, the media were changed to the fresh media with or without TTX at the 0‐min time point. The expression levels of miR‐124 (a) and miR‐9 (b) were assessed 1, 5, 15, 30, 60, and 120 min later by real‐time RT PCR as described in *Materials and Methods. *Cumulative values for miR‐124 and miR‐9 release were calculated as for Figure 7 and are shown in (c). In (a–c), mean ±* SD* of three separate experiments is shown on dotpolts (*n* = 3). In (c), **p < 0.01; unpaired Student's *t* test) [Colour figure can be viewed at wileyonlinelibrary.com]

Because the electric activity of neurons is connected with calcium signaling and secretory activity of these cells (Stewart et al., 2005), we tested whether blocking of electric activity of neurons with TTX would inhibit the accumulation of extracellular miR‐124 and miR‐9. We pre‐treated cells with TTX for 24 hr and changed media 5–6 times to remove miRNA from the surface of the cells. One minute after the last change of media, the levels of miR‐124 and miR‐9 were the same and there was no decrease in neuronal viability (not shown). However, accumulation of miR‐124 (Figure 8a,c) and miR‐9 (Figure 8b,c) was significantly decreased during the 120‐min period in neuronal cells treated with TTX. At the same time, TTX did not decrease intracellular levels of expression of miR‐124 and miR‐9 (not shown). These data indicated that electric activity of neurons stimulated secretion of miR‐124 and miR‐9.

### 3.12 Addition of BSCM or NCM to macrophages results in efficient translocation of miR‐124 from the cell surface to the cytoplasm

**Figure 9:**
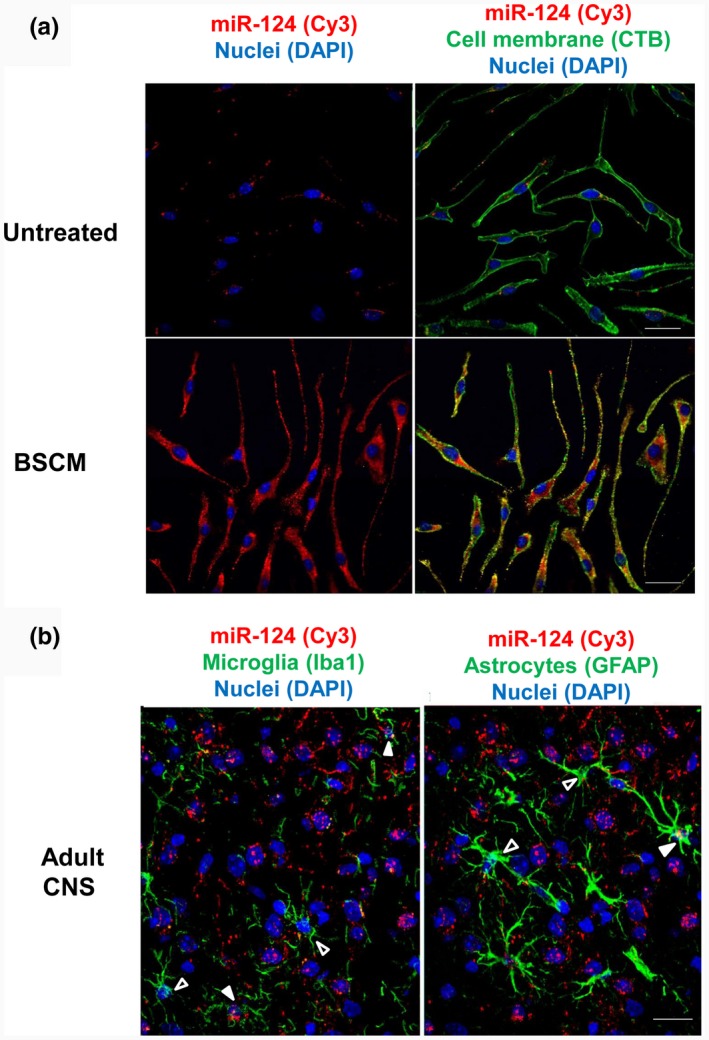
Visualization of miR‐124 in macrophages in vitro and in microglia and astrocytes in vivo in mouse adult brain. Bone marrow‐derived macrophages (BMDMs) were incubated with medium (untreated) or in 50% brain slice conditioned medium (BSCM) for 4 hr as described in *Materials and Methods*. The cells were stained for cell plasma membrane marker GM1 with CTB‐FITC (green) and nuclei with DAPI (blue) as described in *Materials and Methods.* MiR‐124 (red) was detected by fluorescence in situ hybridization as described in *Materials and Methods*. (a) Histology sections were prepared from the normal adult brain of perfused 8‐ to 12‐week‐old B6 female mice as described in *Materials and Methods*. Microglia were stained with Iba1 (green; *left image*), and astroglia were stained with the anti‐GFAP antibody (green; *right image*). MiR‐124 (red) was detected by in situ hybridization as described in *Materials and Methods*. Open arrows indicated microglia and astrocytes without miR‐124 hybridization signal. Filled arrows indicate miR124‐positive microglia (*left image*) or astroglia (*right image*). Nuclei were stained with DAPI (blue). The bar is 10 μm

Finally, we investigated whether neuronal miR‐124 is translocated into the cytoplasm of macrophages and glial cells. We found that addition of BSCM to BMDMs resulted in the accumulation of miR‐124 both on plasma membranes (labeled with CTB, which binds to GM1 ganglioside) and in the cytoplasm of these cells (Figure 9a). At the same time, we did not detect expression of longer transcripts for pre‐miR‐124‐1, pre‐miR‐124‐2, and pre‐miR‐124‐3 after treatment of the cell with BMDMs, indicating that miR‐124 was not transcribed de novo but was instead translocated into the cytoplasm from BSCM.

To identify the similar process in vivo, we visualized miR‐124 in Iba1‐positive microglia and GFAP‐positive astrocytes. We found miR‐124‐positive microglia and miR‐124‐positive astrocytes (Figure 9b, filled arrows) in brain histology sections. MiR‐124‐negative microglia and astrocytes were also present (Figure 9b, open arrow). These data further demonstrated miR‐124 transfer from neuronal cells to astrocytes and neurons.

## 4 DISCUSSION

In this study, we tested our hypothesis that the phenotype of microglia in the CNS is determined by horizontal transfer of neuronal microRNAs from active neurons to microglia. We found that neurons secrete various microRNAs that have the ability to bind and enter the cytoplasm of monocytic cells both in vitro and in vivo. However, in contrast to the generally accepted point of view, most of the neuronal miRNAs were not only associated with exosomes but rather found in extracellular space in multiple forms including complexes with HDLs. Thus, this study highlights an important role of non‐exosomal ECmiRNAs in the communication of neurons with astroglia and microglia, which in turn, determine the phenotype and function of these cells in the CNS. It is especially important in the case of microglia since our study strongly suggest that these cells acquire their deactivated MHC class II^low^CD45^low^ in the CNS microenvironment upon communication with electrically active neurons.

**Figure 10:**
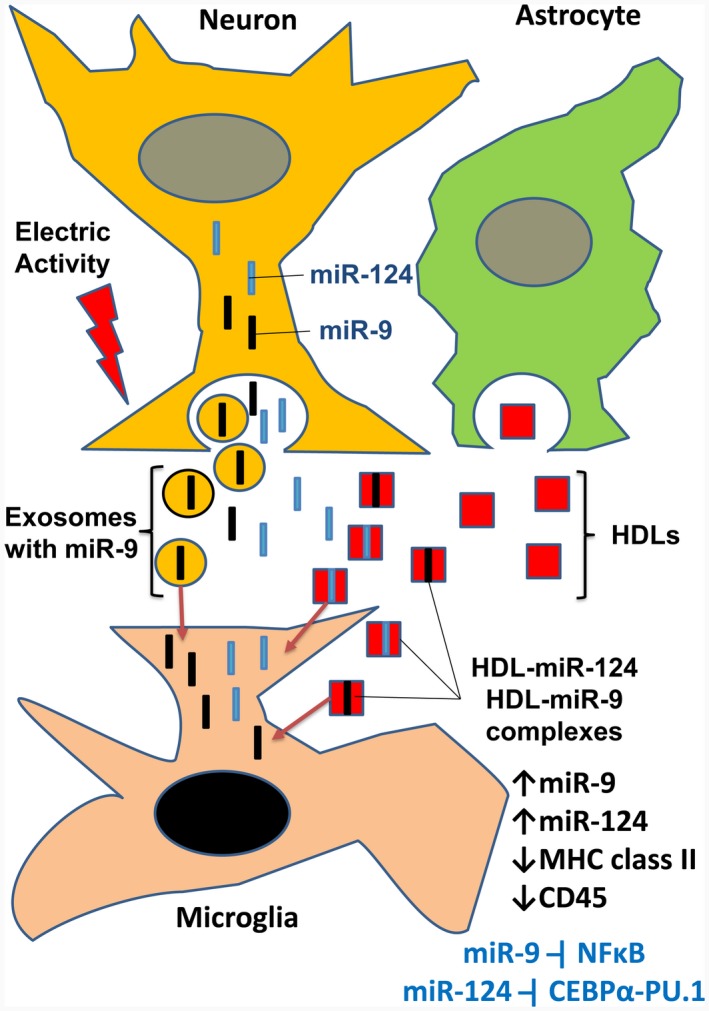
Model of horizontal transfer of miR‐124 and miR‐9 from neurons to microglia. Electrically active neurons secrete exosomes with miR‐9, as well as miR‐124 with miR‐9 in naked form and/or in complex with low molecular weight proteins. Astrocytes secrete HDLs that bind to miR‐124 and miR‐9 in the extracellular space. Exosomes with miR‐9, miR‐124:HDL, and miR‐9:HDL complexes are taken up by microglia. MiR‐9 and miR‐124 are translocated to the cytoplasm of microglia via fusion of miR‐9‐positive exosomes with plasma membrane or transport of HDL‐miR‐124 and HDL‐miR‐9 into the cytoplasm via scavenger receptors. In the cytoplasm, miR‐124 (and possibly miR‐9) deactivate microglia, leading to downregulation of MHC class II and CD45. Inside the cell, miR‐124 are known to inhibit the CEBPα‐PU.1 pathway, whereas miR‐9 inhibit NFκB. Both of these pathways are important for macrophage/microglia activation and expression of activation markers such as MHC class II and CD45

To further understand the mechanism of horizontal transfer of miRNAs from neurons to microglia, we fully characterized neuronal extracellular microRNAs and found that miR‐124, miR‐9, and other neuronal miRNAs are present in the neuronal extracellular space in various forms: exosomes in complex with large protein particles such as HDL, in complex with smaller proteins, and in naked single‐ and double‐stranded forms. MiR‐124 and miR‐9 were constantly released by electrically active neurons, and this process involved mitochondrial activity, calcium signaling, and exocytosis. Extracellular miRNAs miR‐124 and miR‐9 were also present in large quantities on the surface of neuronal cells. These microRNAs were mostly protected from RNase and have the ability to bind to the surface and become internalized by other cell types such as macrophages/microglia. Inside the cell, miR‐124 is known to inhibit the CEBPα‐PU.1 pathway (Ponomarev, Veremeyko, Barteneva et al., 2011), whereas miR‐9 inhibits NFκB in monocytic cells (Bazzoni et al., 2009). Both pathways are important for macrophage/microglia activation and expression of activation markers such as MHC class II and CD45. Thus, horizontal transfer of miR‐124 and miR‐9 from neurons to microglia/macrophages deactivates them in the CNS (Figure 10).

We found that MiR‐9 was the most abundant ECmiRNA in cultured primary neurons. MiR‐9 was found in exosomes and in complex with proteins of smaller molecular weight. The second most abundant ECmiRNA was miR‐124, which was present in NCM in complex with HDLs, but not in exosomes. Prompt release of miR‐124 and miR‐9 was dependent on the electric activity of neurons and could be blocked by tetrodotoxin (TTX). MiR‐124 and miR‐9 were rapidly accumulated in the extracellular space even after several changes of media, and concentrations of these miRNAs were increased after treatment of neuronal cells with β‐galactosidase that digested glycocalyx*,* suggesting large depositions of these microRNAs on the neuronal cell surface associated with glycolipids and/or glycoproteins. Accumulation of miR‐9 and miR‐124 was delayed by treatment with monensin and accelerated after treatment with ionomycin, indicating the importance of the endosomal secretory pathway for secretion of these miRNAs. Microglia did not express miR‐124 and miR‐9 outside of the CNS microenvironment, whereas miR‐124 was detected in the CNS in microglia and astroglia. Thus, our data indicate an important role of extracellular microRNAs miR‐124 and miR‐9 in the communication of neurons and glia.

Our study used small sample size numbers since we used standardized in vitro systems where we combined together cultured cells or brain organotypic cultures isolated from several animals of the same genetic background of an equal/comparable number of animals for both sexes (*n* = 3–6 males and *n* = 3–6 females per experiment). These in vitro systems had low levels of variability and a high level of reproducibility. Indeed for the samples with reverse cycle values (40‐C_T_), the variability was very low with SDs in the range of less than 5% (from 1% to 4%). Therefore, *n* = 3 was sufficient for the standard level of confidence of 95% and statistical significances (Figures 3–4). For the samples with relative levels of miRNA expressions (Figures 1 and 2), SDs were higher than 5% (ranging from 8% to 66%) due to procedures of normalization to snoRNA‐55 for cultured macrophages and possibly other reasons. However, for these samples, the differences for mean values were quite dramatic (from 5 to 20‐fold differences). A similar situation was observed for expressions of MHC class II and CD45 on macrophages with SDs values ranging from 18% to 22% and 2.6–4.7‐fold differences for mean values (Table 2). Therefore sample sizes of *n* = 3–4 were sufficient to observe statistically significant differences among compared groups for most of our experiments. However, it should be kept in mind that for each experiment we combined cultured cells isolated from *n* = 3–6 animals.

Secretion of miRNA into the extracellular space occurs via multiple mechanisms: via formation of exosomes or microparticles, release from necrotic cells, or transport in complex with HDLs (Sohel, 2016). Other mechanisms are also possible for release of naked miRNAs. For example, specific RNA transporters SID‐1 and SID‐2 were found in *C. elegance* and *Drosophila. *SID‐1/2 channel proteins enable systemic RNA interference and transfer of dsRNA from one cell to another, with SID‐1 mediating release and SID‐2 mediating uptake of regulatory RNA (McEwan, Weisman, & Hunter, 2012; Shih, Fitzgerald, Sutherlin, & Hunter, 2009). Similar transporters with similar functions were recently found in humans and mice (Elhassan, Christie, & Duxbury, 2012; Nguyen et al., 2017). Our data indicate that miRNAs are released from neurons not in the context of microparticles, in that filtration through a 100‐nm filter did not decrease expression of most neuronal (miR‐124, miR‐9, miR‐9*, miR‐186) and other miRNAs (miR‐155). Filtration of NCM through a 25‐nm filter significantly decreased expression of miR‐155 and, to a lesser extent, miR‐9. By isolation of exosomes, we found that miR‐9 and miR‐155 were enriched in exosomes, along with miR‐9* and miR‐124*. On the other hand, almost all miR‐124 and a large proportion of miR‐9, along with miR‐124* and miR‐9*, were not associated with exosomes. Our analysis demonstrated that in the fraction with molecular weight of >100 kDa, miR‐124 and miR‐9 were associated with HDLs. It is interesting that neurons do not produce their own HDLs, but these large protein complexes are produced by glial cells, which are present in neuronal cultures (Vitali et al., 2014). This fact suggests that miR‐124 and miR‐9 were probably secreted by neurons not in complex with HDLs but in complexes with other smaller proteins and/or in naked single‐ and double‐stranded forms. These neuronal ECmiRNAs most likely bind to glial HDLs in extracellular space. We found quite large proportions of miRNA in the low molecular weight fraction of 10–30 kDa of NCM. Moreover, experiments with treatment of the 10–30‐kDa fraction with proteinase and filtration demonstrate the presence of both single‐ and double‐stranded forms in miR‐9 and double‐stranded form of miR‐124. It is remains enigmatic how miRNA is secreted by live neuronal cells not in complex with exosomes or microparticles. Our data suggest that the mitochondrial inhibitor NaN_3_ and protein transport inhibitor monensin inhibited short‐term release but did not significantly affect long‐term cumulative release of miR‐9 and miR‐124, whereas ionomycin induced calcium influx in neuronal cells and intensified this process. These data indicate that the secretion of miRNA depended on exocytosis, which in turn was stimulated by calcium signaling. Our data are in good agreement with previously published studies (Kuwabara, Hsieh, Nakashima, Taira, & Gage, 2004; Sohel, 2016; Zhang et al., 2015), showing the importance of all these processes for ECmiRNA release. We also found that production of miR‐124 and miR‐9 were substantially decreased by TTX. This finding implies that spontaneous electric activity of neurons was important for miRNA secretion. Electric activity of neurons is known to increase exocytosis (Chivet et al., 2012; Park, 2017), and also facilitate opening of voltage‐gated channel proteins that might also directly and/or indirectly contribute to the release of ECmiRNAs. This is quite an interesting and important finding, which will be further investigated in the future.

We found that ECmiRNA were protected from RNase. This indicates that miRNAs either exist in complexes with proteins or have a specialized conformation that is resistant to RNase. Previous studies also indicated that ECmiRNAs were RNase insensitive (Wang et al., 2010). However, we also found that a large proportion of miRNAs were bound to the cell surface. Moreover, the cleavage of carbohydrates from cell surfaces with β‐galactosidase enhanced the release of miR‐124 and miR‐9 into the extracellular space. We believe that binding of miRNA to cell surface glycolipids or glycoproteins may also contribute to their protection from RNase.

To exert intercellular communication, miRNAs have to bind to the surface of the recipient cell and be internalized into the cytoplasm. The internalization of miRNA occurs via multiple mechanisms. The first mechanism involves fusion of exosomes and microparticles with the plasma membrane. The second mechanism involves endocytosis of miRNA bound to the plasma membrane, and the third mechanism implies uptake of miRNA:HDL complexes via the scavenger receptor SR‐BI. Our data demonstrate that the fraction >100 kDa was enriched with miRNA‐124 and miR‐9‐HDL complexes. This fraction >100 kDa resulted in upregulation of miR‐124 in macrophages. Given that scavenger receptors SR‐BI are expressed in macrophages, microglia, and astrocytes (Husemann & Silverstein, 2001), we believe that it is the main pathway of miR‐124 internalization in microglia and astrocytes in vivo*.* Our in vitro experiments show that miRNA is co‐localized with GM1‐positive lipid rafts in the surface of macrophages and is also found in the cytoplasm. Therefore, there is also the possibility that the 10–100‐kDa fraction of NCM with miRNA‐124 could also bind to macrophage surfaces and become internalized via endocytosis or phagocytosis.

The upregulation of miR‐124 in the cytoplasm of macrophages after addition of NCM could occur through two chief mechanisms: 1) internalization of exogenous miR‐124 from the extracellular space and the cell surface into the cytoplasm, and 2) induction of endogenous miR‐124 in macrophages. Indeed, very low levels of miR‐124 were found in the nuclei of macrophages, indicating the possibility of upregulation of miR‐124 on a transcriptional level. However, we could not detect expression of any of 3 transcripts for miR‐124 precursors (pre‐miR‐124.1, pre‐miR‐124.2, and pre‐miR‐124.3) after co‐incubation with NCM or BSCM (not shown). At the same time, our previous study demonstrated that the expression of pre‐miR‐124 transcripts was induced by IL‐4 and IL‐13 in macrophages (Veremeyko et al., 2013). However, when we used IL‐4‐ or IL‐4/13R‐deficient animals, the level of expression of miR‐124 in microglia in vivo remained unaltered (Veremeyko et al., 2013). This result indicates that miR‐124 is probably translocated from neurons to glial cells including microglia and astrocytes rather than being induced on a transcriptional level. This impression is confirmed by the finding that pre‐miR‐124 transcripts were also not detected in ex‐vivo isolated microglia (not published). At the same time, we demonstrated here the localization of miR‐124 in Iba1‐positive microglia and GFAP‐positive astrocytes. Taken together, these findings favor our hypothesis of horizontal transfer of miR‐124 from neurons to glia.

Several studies suggest the importance of miRNA in deactivation of microglia and macrophages. We demonstrated that overexpression of miR‐124 decreased M1 macrophage activation and promoted the M2 regulatory phenotype, whereas miR‐124 inhibitors increase the level of M1 activation (Ponomarev, Veremeyko, Barteneva et al., 2011; Veremeyko et al., 2013). We also found that miR‐124 deactivated microglia in the normal CNS (Ponomarev, Veremeyko, Barteneva et al., 2011), whereas knockout of DICER (the enzyme responsible for processing of pre‐miRNA precursor molecules into the shorter form of functional miRNAs) resulted in elevated levels of activation of microglia in vivo (Varol et al., 2017). However, the absence of DICER in adult microglia did not result in a substantial level of spontaneous activation of these cells in the CNS in terms of upregulation of CD45 and MHC class II. Our study suggests that the role of microRNAs might be underestimated in DICER‐deficient mice, in that the expression of miRNA in microglia could also be induced by horizontal transfer of microRNAs from neurons. Thus, our study highlights the importance of neuron‐microglia communication in the maintenance of the deactivated phenotype of microglia in the normal CNS. Communications between neurons and microglia/astrocytes were recently shown to be important for neuronal development and repair after injury, whereas signals from neuronal cells to microglia/astrocytes remain greatly understudied (Reemst, Noctor, Lucassen, & Hol, 2016; Szalay et al., 2016). We demonstrated the important role of miRNA‐124 and miR‐9 in this process, which would have applications for the development of future approaches to modulate microglial cell activation during neuroinflammatory and neurodegenerative diseases.

## CONFLICT OF INTEREST

The authors declare no conflicts of interest.

## AUTHOR CONTRIBUTIONS

All authors had full access to all the data in the study and take responsibility for the integrity of the data and the accuracy of the data analysis. *Conceptualization*, T.V. and E.P.; *Methodology*, T.V., I.K., and E.P.; *Investigation*, T.V., I.K., M.D., A.Y., E.K., and E.P.; *Formal Analysis*, T.V., I.K., M.D., and E.P.; *Resources*, N.B.; *Data Curation*, T.V. and E.P.; *Writing—Original Draft*, T.V., N.B., and E.P.; *Writing—Review & Editing*, T.V. and E.P.; *Visualization*, E.P.; *Supervision*, E.P.; *Project Administration*, A.Y. and E.P.; *Funding Acquisition*, E.P.

## Data Availability

All the data and materials associated with the manuscript will be available to other researchers upon the reasonable request.
